# CD81 and CD82 expressing tumor-infiltrating lymphocytes in the NSCLC tumor microenvironment play a crucial role in T-cell activation and cytokine production

**DOI:** 10.3389/fimmu.2024.1336246

**Published:** 2024-03-07

**Authors:** Kwangmin Na, Seul Lee, Dong Kwon Kim, Young Seob Kim, Joon Yeon Hwang, Seong-san Kang, Sujeong Baek, Chai Young Lee, Seung Min Yang, Yu Jin Han, Mi hyun Kim, Heekyung Han, Youngtaek Kim, Jae Hwan Kim, Seunghyun Jeon, Youngseon Byeon, Jii Bum Lee, Sun Min Lim, Min Hee Hong, Kyoung-Ho Pyo, Byoung Chul Cho

**Affiliations:** ^1^ Severance Biomedical Science Institute, Yonsei University College of Medicine, Seoul, Republic of Korea; ^2^ Brain Korea 21 PLUS Project for Medical Science, College of Medicine, Yonsei University, Seoul, Republic of Korea; ^3^ Yonsei New Il Han Institute for Integrative Lung Cancer Research, Yonsei University College of Medicine, Seoul, Republic of Korea; ^4^ JEUK Institute for Cancer Research, JEUK Co., Ltd., Gumi-City, Republic of Korea; ^5^ Division of Medical Oncology, Yonsei Cancer Center, Yonsei University College of Medicine, Seoul, Republic of Korea

**Keywords:** tetraspanins, immunotherapy, tumor-infiltrating lymphocyte, cell therapy, T lymphocyte

## Abstract

**Introduction:**

To understand the immune system within the tumor microenvironment (TME) of non-small cell lung cancer (NSCLC), it is crucial to elucidate the characteristics of molecules associated with T cell activation.

**Methods:**

We conducted an in-depth analysis using single-cell RNA sequencing data obtained from tissue samples of 19 NSCLC patients. T cells were classified based on the Tumor Proportion Score (TPS) within the tumor region, and molecular markers associated with activation and exhaustion were analyzed in T cells from high TPS areas.

**Results:**

Notably, tetraspanins CD81 and CD82, belonging to the tetraspanin protein family, were found to be expressed in activated T cells, particularly in cytotoxic T cells. These tetraspanins showed strong correlations with activation and exhaustion markers. *In vitro* experiments confirmed increased expression of CD81 and CD82 in IL-2-stimulated T cells. T cells were categorized into CD81^high^CD82^high^ and CD81^low^CD82^low^ groups based on their expression levels, with CD81^high^CD82^high^ T cells exhibiting elevated activation markers such as CD25 and CD69 compared to CD81^low^CD82^low^ T cells. This trend was consistent across CD3^+^, CD8^+^, and CD4^+^ T cell subsets. Moreover, CD81^high^CD82^high^ T cells, when stimulated with anti-CD3, demonstrated enhanced secretion of cytokines such as IFN-γ, TNF-α, and IL-2, along with an increase in the proportion of memory T cells. Bulk RNA sequencing results after sorting CD81^high^CD82^high^ and CD81^low^CD82^low^ T cells consistently supported the roles of CD81 and CD82. Experiments with overexpressed CD81 and CD82 showed increased cytotoxicity against target cells.

**Discussion:**

These findings highlight the multifaceted roles of CD81 and CD82 in T cell activation, cytokine production, memory subset accumulation, and target cell cytolysis. Therefore, these findings suggest the potential of CD81 and CD82 as promising candidates for co-stimulatory molecules in immune therapeutic strategies for cancer treatment within the intricate TME.

## 1 Introduction

In the context of T cells, co-stimulatory molecules, such as Cluster of Differentiation 28 (CD28) and 4-1BB (CD137), play a pivotal role not only in their activation but also in regulating immune suppression. These co-stimulatory molecules can enhance the secretion of various cytokines and promote the expression of their corresponding receptors (1). These processes are fundamental for T-cell activation, proliferation, and induction of T cells into diverse functional subgroups (2–5).

The definition of T-cell co-stimulation has been continually evolving, driven by the discovery of new co-stimulatory receptors, biochemical characterization of their downstream signaling events, and clarification of their immunological functions (3). In this context, several therapeutic applications have been investigated (6), including the potential application of various co-stimulatory molecules in the field of Chimeric Antigen Receptor (CAR) development (7). Additionally, T-cell bispecific antibodies, which act as tumor-targeting 4-1BB agonists, have been explored as potential combination therapies (8). For targeting 4-1BB, both agonistic anti-human 4-1BB antibodies (9–12), and second-/third-generation 4-1BB/CD3ζ CAR-T cells have been investigated (13–15). Notably, the agonistic anti-human 4-1BB antibody, urelumab (BMS-663513), a human IgG4 antibody (anti–hu4-1BB huIgG4), has shown dose-dependent hepatotoxicity in patients, possibly attributed to the cross-linking of 4-1BB via FcγRIIb-expressing liver-resident cells, including hepatic myeloid and sinusoidal endothelial cells (16, 17).

Ongoing research on co-stimulation has made significant progress, leading to the discovery of numerous novel molecules through systematic examinations (18). However, the target co-stimulatory molecule should be selected with careful consideration as the function of co-stimulation varies among T-cell subsets. For instance, recent research revealed that human memory T cells exhibit greater sensitivity to the effects of CD28 co-stimulation than naive T cells (19).

Recently, a novel molecular mechanism involving tetraspanins was reported. Emerging evidence suggests that tetraspanins contribute to the stability of surface proteins and play regulatory roles in signal transduction. Moreover, tetraspanins affect the functional aspects of immune activators expressed on immune cell surface (20). However, their distribution and role in T cells in the TME has remained unknown. In this study, we examined the distribution and expression patterns of various tetraspanins in the lung cancer TME.

Based on the data obtained from single-cell RNA sequencing of clinical samples, we confirmed that tetraspanins play a crucial role in T-cell activation. Subsequently, we further validated this through *in vitro* experiments and transcriptome data. Our findings provide valuable insights into the potential applications of tetraspanins in the field of cancer immunotherapy.

## 2 Materials and methods

### 2.1 Patients and samples

Lung cancer tissues from patients with non-small cell lung cancer (NSCLC), aged 51-80 years, belonging to both sexes were used in this study The study protocol has been approved by the Institutional Review Board of Severance Hospital (IRB) under study numbers 4-2016-0788 and 4-2014-0775.

### 2.2 Preparation of NSCLC tumor samples

Briefly, NSCLC tissues were obtained from 19 patients who were operated at the Yonsei Cancer Center at Yonsei University. Written informed consent for tissue and clinical information was obtained from all patients. Information related to standard clinicopathological variables including sex, age, tumor site, disease state, type of procedure, tumor state (TNM classification), and treatment type was collected from each patient as part of a prospective NSCLC database.

### 2.3 Single-cell RNA library generation and sequencing

Single-cell suspensions were processed using 10x Genomics. The libraries were prepared using Chromium Single Cell 5’ Reagent Kits (v2) and Single Cell 5’ Library & Gel Bead Kit (10x genomics, Pleasanton, CA, USA) followed by the Single-Cell 5′ Reagent Kits (v2). The DNA libraries were run on a NovaSeq 6000 system (Illumina, San Diego, CA, USA). Sequencing results were de-multiplexed and converted to the FASTQ format using Illumina software and the 10x Genomics Cell **Ranger** 6.1. The cDNA insert was aligned with the hg38/GRCh38 reference genome. Only confidently mapped data was subjected to further analysis, including identification of highly variable genes, dimensionality reduction, standard unsupervised clustering algorithm application, and identification of differentially expressed genes using Seurat (version 4.9.9). Raw data were initially filtered to remove **doublets** using Scrublet (version 0.2.3, GitHub), and only high-quality cells were finally retained. Cells expressing less than 200 genes, genes expressed in less than 3 cells, and cells with more than 20% fractions of mitochondrial counts were also discarded. Additionally, cells were filtered by each sample with a minimum of 30 cells. To visualize the data, the dimensionality of the scaled integrated data matrix was further reduced to project the cells onto a two-dimensional space using principal component analysis. To identify cells comprising the TME based on single-cell RNA sequence (scRNAseq.), we implemented Azimuth annotation based on SCT-transform. For this purpose, we utilized the ‘annotation.l1’ from ‘human-lung v1’ as a reference to distinguish 54 cell types (Azimuth version 0.4.3). Cells were clustered into six categories: T cell/NK cells, B cells, macrophages, mast cells, endothelial cells, and epithelial cells. To analyze tumor-infiltrating lymphocytes (TILs), we examined effector/memory T-cell and naive T-cell subsets within the T-cell/NK cell cluster.

### 2.4 Isolation of human T cells from PBMC

PBMCs from healthy donors samples were isolated using Ficoll-Hypaque gradient centrifugation. T cells were obtained using SepMate™ PBMC Isolation Tube (STEMCELL, Vancouver, Canada). Isolated T lymphocytes were maintained in RPMI 1640 or ImmunoCult™-XF T cell Expansion Media (STEMCELL, Vancouver, Canada) with 50 U/ml interleukin-2 (PeproTech, Cranbury, NJ, USA). CD3^+^ T cells, CD4^+^ T cells, and CD8^+^ T cells were isolated using Pan T negative selection kit (Miltenyi Biotec, North Rhine-Westphalia, Germany), CD4^+^ T cell negative selection kit (Miltenyi Biotec, North Rhine-Westphalia, Germany) and CD8^+^ T-cell negative selection kit (Miltenyi Biotec, North Rhine-Westphalia, Germany), respectively, following the manufacturer’s instructions. Isolated T cells were activated by adding a CD3/28 T-cell activator (STEMCELL, Vancouver, Canada) and 50 U/ml interleukin-2. T cells were resuspended in CryoStor CS10(STEMCELL, Vancouver, Canada) at -80°C and thawed quickly in a 37°C water bath. All cells were grown in a humidified incubator at 37°C supplied with 5% CO_2_ and tested regularly for Mycoplasma contamination.

### 2.5 T-cell sorting for *in vitro* assay

T cells were activated with an anti-CD3/CD28 T-cell activator and IL-2 for 2 days prior to sorting. Following activation, cells were stained with anti-CD81 and anti-CD82 antibodies for gating. Sorting was performed using a MACSQuant Tyto instrument (Miltenyi Biotec, Bergisch Gladbach, Germany) by dividing the cells into CD81^high^CD82^high^ and CD81^low^CD82^low^ populations. Subsequently, the sorted cells were seeded onto a plate pre-coated with anti-CD3 (0.5 µg/ml), incubated under standard culture conditions, and both cells and supernatants were harvested at the end of 2 days.

### 2.6 Measurement of CD markers for activation and exhaustion on T cells

To analyze the expression of memory markers and assess T-cell activation within the immunological synapse, T cells were harvested immediately prior to the addition of IL-2 on Day 0, Day 2, Day 5, and Day 10. T cells were collected daily until day three to analyze T-cell receptor (TCR) signaling cascade using the following antibodies: anti-CD3 (300434, BioLegend), anti-CD4 (300560, BioLegend), anti-CD8 (301008 and 301056, BioLegend), anti-PD-1 (329908, BioLegend), anti-LAG-3 (369312, BioLegend), anti-CD25 (302622, BioLegend), anti-CD69 (310930, BioLegend), anti-CD81 (349518, 349504, BioLegend), anti-CD82 (342114, BioLegend), anti-CD45RA (304160, BioLegend), anti-CCR7 (353214, BioLegend), anti-STAT5 phospho (Tyr694)(936904, BioLegend), and anti-ERK1/2 phospho (Tyr204)(369508, BioLegend) antibodies. To prepare the staining solution, the antibodies were resuspended in 100 μl fluorescence-activated cell sorting (FACS) buffer including 1% BSA in Dulbecco’s phosphate-buffered saline (DPBS). Staining was performed for 20 min at 4°C in the dark. Subsequently, the cells were washed with 2 ml FACS buffer, resuspended, and analyzed. For staining STAT5 phospho and ERK1/2 phospho, T cells were fixed and permeabilized using 200 μl True-Nuclear Transcription Factor Perm/Fix (424401, BioLegend) for 20 minutes at 4°C. The cells were then washed with 2 ml of intracellular staining buffer containing 1% BSA; 0.1% sodium azide; and 0.1% saponin in DPBS before being analyzed. Multicolor flow cytometry analysis was performed on a BD LSR-fortessa™ X-20 instrument (BD Bioscience, Franklin Lakes, NJ, USA) and data were acquired and analyzed using FlowJo v10 (Tree Star, Ashland, OR, USA).

### 2.7 Measurement of cytokines and chemokines

To assess cytokine and chemokine production, non-activated T cells (not treated with CD3/28 stimulator or IL-2) were used. For preparing the antibody pre-coated plate, the following antibodies with indicated concentrations were used: anti-CD3 (14-0037-82, Invitrogen; 0.5 μg/mL), anti-CD81 (349502, BioLegend; 5 μg/mL), and anti-CD82 (342102, BioLegend; 5 μg/mL). These antibodies were resuspended in DPBS and coated onto the plate at 4°C, 24 hours before use. For RNA sequencing (RNA-seq), cells were harvested on Day 1. For cytokine analysis and flow cytometry, the cells were harvested on Day 2. To determine the concentrations of IFN-γ, TNF-α, and IL-2 in the T-cell supernatant, we used the following commercially available ELISA kits: Human IFN-γ ELISA kit (EHIFNG, Invitrogen), TNF-α ELISA kit (KHC3011, Invitrogen), and IL-2 ELISA kit (BMS221, Invitrogen). Additionally, we used the Human XL Cytokine Array (ARY022B, R&D Systems) to assess the concentration of various cytokines in the T-cell supernatant after they were cultured for either 2 or 3 days.

### 28 Bulk RNA librarying and sequencing

Briefly, the processing of FFPE tumor samples was outsourced to MACROGEN (SEL, South Korea) and RNA sequencing data were obtained. Total RNA extracted from the FFPE samples was processed to prepare an mRNA-sequencing library using the SureSelectXT RNA Direct Library Preparation kit (Illumina, San Diego, California, USA) according to the manufacturer’s instructions. All samples were sequenced on an Illumina sequencer using paired-end 100 bp reads. Raw image data were transformed into sequence data by base-calling and stored in FastQC (v0.11.7) format. Paired-end reads of 14 independent samples were trimmed using Trimmomatic (v0.38; http://www.usadellab.org/cms/page=trimmomatic). The trimmed reads were aligned using Bowtie2 (v2.3.4.1) and mapped to the reference mouse genome using HISAT2 (v2.1.0). Gene-level read counts were generated using the StringTie software (v2.1.3b). Genes showing significant differential expression were determined using the DESeq2 package (version 1.26). All gene sets in V.7.0, of the Molecular Signatures Database were analyzed using V.4.0.3 of Gene Set Enrichment Analysis (GSEA) and corrected for multiple hypothesis testing. The p-value threshold was set at 0.05. Heatmaps of differentially expressed genes were generated using Prism (version 9.0). High-depth RNA sequencing was performed to analyze the TCR repertoire. TCR sequences were extracted by V.3.0 of MiXCR and analyzed using the TcR package (V.2.2.4.1).

### 2.9 CD81 and CD82 overexpression in Jurkat cells

CD81 and CD82 transgenes were inserted into multiple cloning sites of CMV promoter-containing vectors. Large-scale production of CD81 and CD82 was achieved by transient co-transfection of Lenti-293T cells using Lipofectamine 3000 (Invitrogen, Waltham, Massachusetts, USA). Lentivirus batches were ultracentrifuged at 4,000 x *g* at 4°C for 15 min using Amicon Ultra-15(Millipore, Ireland). Lentiviral titers were determined by assessing the viral p24 antigen concentration using ELISA (Clontech, Palo Alto, CA, USA). Hereafter, it is expressed in milligram of p24 equivalent units per milliliter. The cells were treated with a Multiplicity of Infection of two and cultured in 24-well plates. Fresh medium was added every 2 days. To evaluate the transduction efficiency, T cells were harvested on Day 5 following transduction.

### 2.10 Cytotoxicity analysis of bispecific T-cell engager

We performed high-throughput and quantitative real-time cell analyses using the xCELLigence RTCA HT system from Agilent Technologies (Santa Clara, CA, USA) to measure the cytotoxicity of CD81 transduced T cells and CD82 transduced T cells. Target gene-expressing CHO cells were seeded (1 × 10^4^ cells/well) in disposable xCELLigence gold-coated E-plates and the seeded cells were allowed to adhere and proliferate for 24 h. Next, effector cells were seeded (5 × 10^4^ cells) and an anti-CD3 × anti-CEA bispecific antibody (25 nM) was added to each well. The xCELLigence system converted electrical impedance into cell-index data, which were recorded at 15 min intervals throughout the experiment. This allowed for the continuous monitoring of cell behavior and cytotoxicity. Following experiment completion, the data were analyzed using Agilent Technologies, Inc. RTCA Software Pro 2.6.0. A cell index plot was generated for each sample well and compared with that of the control wells for further analysis and interpretation of the cytotoxicity results.

### 2.11 Statistical analysis

Two-way analysis of variance, followed by Tukey’s *post hoc* test, was used to compare data from two or more groups using GraphPad Prism software (version 6.0; San Diego, CA, USA). Results with *P* < 0.05 were considered statistically significant.

## 3 Results

### 3.1 CD81 and CD82 expressed in the human lung TME are associated with T-cell activation (single-cell RNA sequencing DATA)

**Figure 1:**
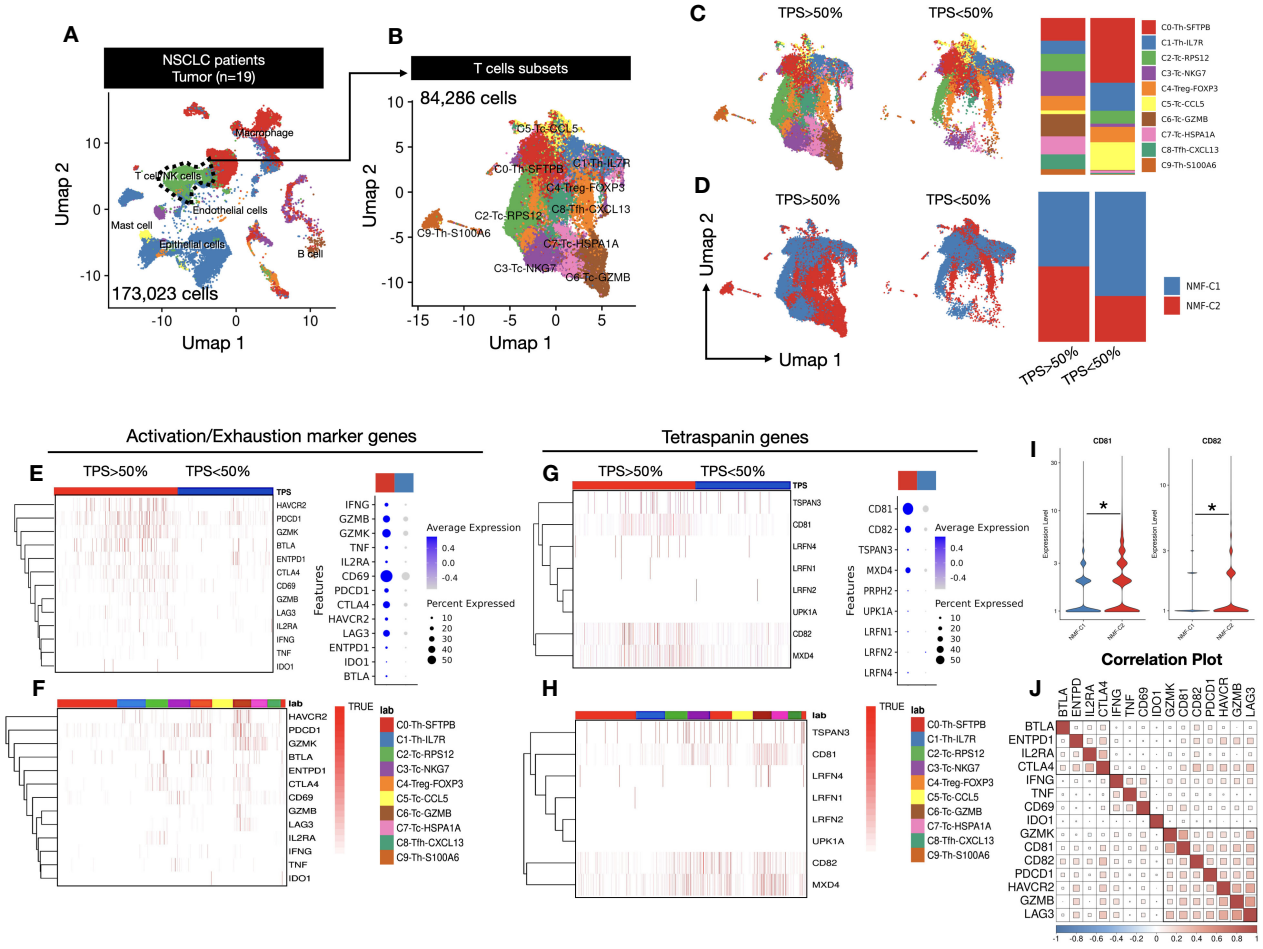
**(A)** UMAP1-UMAP2 dimension plot visualizing a complex cellular landscape with 173,023 cells from 19 patients, focusing on T and NK cells, highlighted with a green cluster outlined using a dashed line. **(B)** UMAP1-UMAP2 Dimension plot closely examining cell clusters derived from T and NK cells identified in **(A)**. A total of 84,286 cells were examined across 10 distinct clusters, providing insights into the diverse T and NK cell subtypes. **(C)** TPS-dependent gene expression transition exploring gene expression within T cells, considering the influence of tumor proportion score (TPS). Cells are categorized based on TPSs (below and above 50%), with a bar plot illustrating cluster proportions. **(D)** NMF cluster analysis revealing re-clustering of T cells into NMF1 and NMF2, based on Supplementary Figure 2C, demonstrating the spatial distribution of TPSs above and below 50% among T-cell subsets. Bar plot quantifies the TPS categories within NMF-defined clusters. **(E)** Activation and exhaustion marker heatmap assessing the activation and exhaustion status by reflecting gene enrichment in activated T cells. Dot plot indicates the expression percentages of these genes in a TPS-dependent manner. **(F)** Activation and exhaustion marker heatmap in T-cell clusters within the 10 T-cell clusters from **(B)**, illustrating activation and exhaustion marker enrichment, elucidating their distribution across diverse T-cell subtypes. **(G)** Tetraspanin gene expression heatmap focusing on eight selected genes among 33 tetraspanins, showcasing their expression percentages within the cell population. **(H)** Tetraspanin gene expression in T-cell clusters similar to **(F)**, dissecting tetraspanin gene enrichment within the 10 T-cell clusters, highlighting differential expression patterns. **(I)** CD81 and CD82 expression violin plot emphasizing the expression levels of CD81 and CD82 genes in relation to NMF clustering, providing insights into their roles in the T-cell response. Significance was evaluated using t-tests (*p ≤ 0.05). **(J)** Correlation plot revealing intricate relationships between activation markers, exhaustion markers, and CD81 and CD82 genes. Three major clusters were identified with distinct correlation patterns.

We analyzed single-cell RNA sequencing (scRNA-seq) data from tissue samples of 19 patients with NSCLC. The tissues were derived from surgical specimens of patients with stage 1-2 cancer. A total of 173,023 cells were analyzed, and we classified them into six distinct clusters, namely macrophages, T cells/NK cells, endothelial cells, B cells, mast cells, and epithelial cells (**Figure 1A**). Furthermore, T cells/NK cells subset were sub-clustered using Azimuth annotation into effector, memory, and naive T-cell subtypes. We identified 10 subclusters using an unbiased clustering approach, with a total of 84,286 cells (**Figure 1B**). Cell annotation was performed based on the top gene markers, and annotations for helper T cells, cytotoxic T cells, regulatory T cells, and follicular helper T cells were identified (**Supplementary Figure 1A**). These T cells were further categorized based on the TPS of the primary tumor region. The TPS is an indicator of PD-L1 expression levels, where higher PD-L1 expression levels correlate with a greater number of T cells within a tumor. We visualized the relationship between each of the 10 subclusters and TPS data through bar plots and dimension plots, which revealed a clear trend of increased number of specific clusters with increase in TPSs and a decreased number of clusters with low TPSs (**Figure 1C**, **Supplementary Figure 1C**). Additionally, we examined using NMF (Non-negative Matrix Factorization) analysis the changes in the expression patterns associated with TPSs (**Figure 1D**, **Supplementary Figure 2A**). The division of high-scoring k-means within the consensus plot was markedly significant (**Supplementary Figure 2B**). Consequently, we divided the dataset into two clusters using NMF analysis (**Supplementary Figure 2C**). We observed that NMF cluster2 exhibited high expression levels when the TPS was high, whereas NMF cluster1 displayed high expression levels when the TPS was low (**Figure 1D**, **Supplementary Figure 2D**).

We assessed the expression levels of typical activation and exhaustion markers within each cluster to examine their correlation with classified T-cell subsets and presented the findings in a heatmap. Notably, both activation and exhaustion markers were highly expressed in response to high TPS. Activation markers, including IFNG, GZMB, and CD69, were highly expressed in clusters with high TPSs; exhaustion markers such as PDCD1, CTLA4, and LAG3 also demonstrated high expression in clusters with high TPSs, as shown in the dot plot (**Figure 1E**). To identify the cell types with increased expression of these genes. We examined the classified cell types (**Supplementary Figure 1**) to identify the cell types with increased gene expression. We observed that these genes were highly expressed in cluster6, comprising cytotoxic cells with high GZMB expression. This observation led us to hypothesize that activating T cells were enriched within cluster6 in the TME (**Figure 1F**). Next, to examine the correlation between TPSs and tetraspanin expression, we selected 8 of 33 tetraspanins belonging to the human tetraspanin family. Our findings revealed that CD81 and CD82 were highly expressed in T cells, and there was a general tendency for tetraspanin gene expression levels to increase with higher TPSs. Among these, CD81 and CD82 exhibited the most dramatic differences in expression (**Figure 1G**). Subsequently, we explored the cell types in which this increased expression occurred, and found that the expression was elevated in T-cell types other than cytotoxic T cells. (**Figure 1H**).

Therefore, we examined the overall expression based on the NMF criteria without specifying T-cell types and found that NMF cluster2, associated with higher TPSs, displayed an increasing trend (**Figure 1I**). Additionally, we examined the correlation between CD81 and CD82 and activation and exhaustion markers. The results indicated that all the genes had a positive correlation and could be broadly divided into three clusters. Clusters containing CD81 and CD82 were identified as cluster 2 and exhibited a notably high correlation with PDCD1, GZMB, and LAG3. LAG3 and PDCD1 are considered early exhaustion genes that showed a relatively high expression tendency upon activation. Based on these data, we not only observed a close association between CD81 and CD82 but also suggested that these two molecules have a strong correlation with activation and exhaustion markers in T cells, potentially acting as regulatory molecules for T-cell activation (**Figure 1J**).

### 3.2 T cells expressing high levels of CD81 and CD82 also exhibit increased expression of activation markers

CD81 and CD82 expression in activated T cells correspond to the presence of activation and exhaustion markers. Thus, we assessed the expression levels of CD81 and CD82 after a 2-day stimulation with IL-2R using 50 U/mL of IL-2. These molecules exhibited a broad range of expression levels in T cells, regardless of IL-2R stimulation; however, their expression increased considerably upon IL-2R stimulation. The expression of CD81 in CD3^+^ and CD8^+^ T cells after stimulation with IL-2R was 1.25-fold higher than that without stimulation. Similarly, CD82 in CD3^+^ and CD8^+^ T cells exhibited a 1.5-fold increase in expression upon stimulation compared with that without stimulation. In CD4^+^ T cells, both CD81 and CD82 showed higher expression levels with stimulation than without, but these differences were not statistically significant (**Supplementary Figure 3A**).

**Figure 2:**
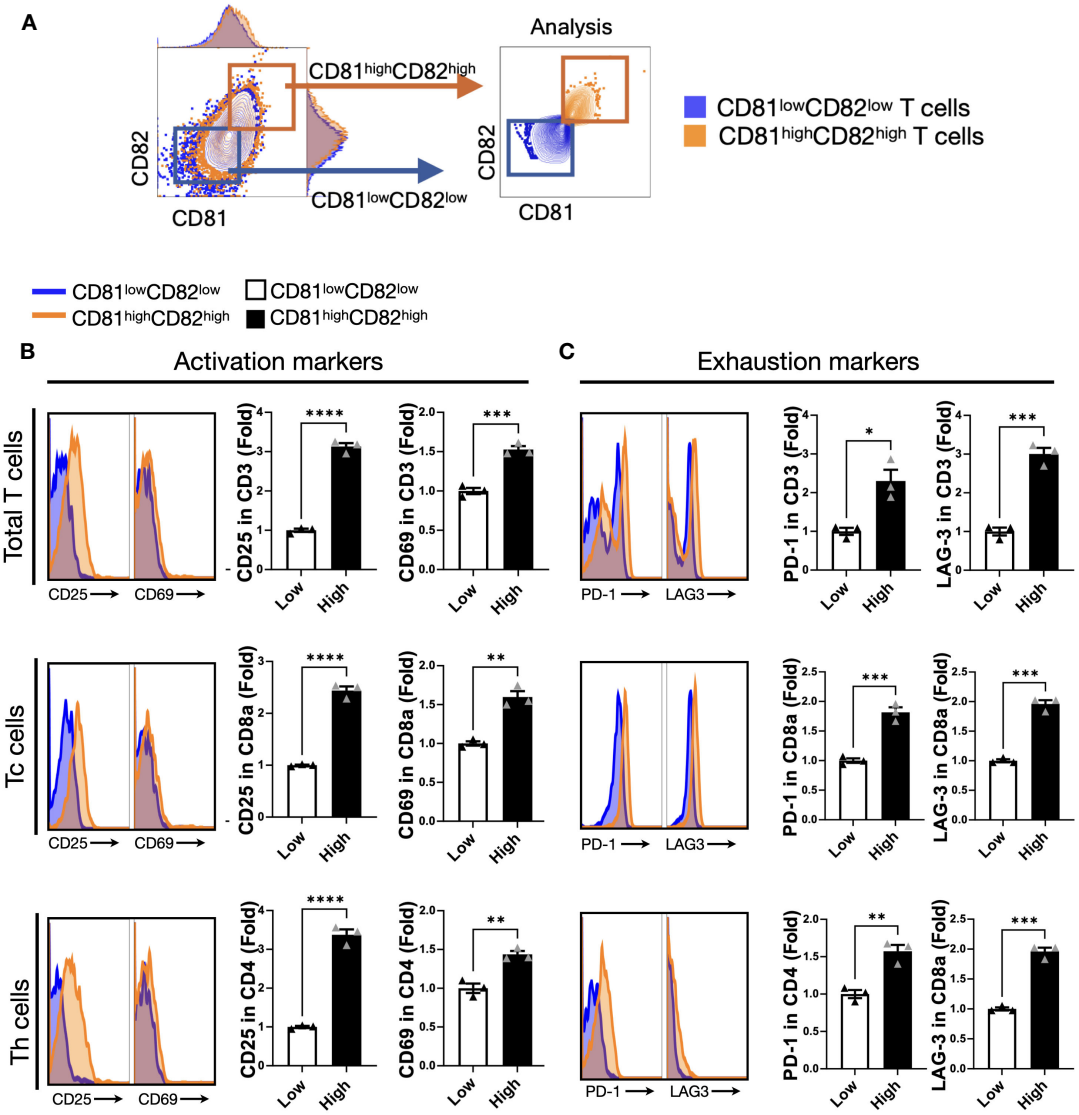
T cells displaying heightened levels of CD81 and CD82 expression demonstrate higher levels of activation markers. **(A)** T cells were stratified into CD81^low^CD82^low^ and CD81^high^CD82^high^ subsets. The blue square denotes CD81^low^CD82^low^, whereas the orange square indicates CD81^high^CD82^high^. **(B)** Comparison of the expression levels of activation markers, CD25 and CD69, within the CD81^low^CD82^low^ and CD81^high^CD82^high^ regions across the total number of CD3^+^ T, CD8^+^ T, and CD4^+^ T cells. Blue region signifies gating for low CD81 and CD82 expression, whereas the orange region designates gating for the simultaneous high expression of CD81 and CD82. This comparison is presented as a histogram and bar graph to display fold changes. In the bar graph, mean fluorescence intensity (MFI) values under baseline conditions are represented using white bars, and MFI values following IL-2 treatment are indicated using black bars. Data is derived from three independent wells. **(C)** Comparison of the expression of exhaustion markers PD-1 and LAG-3 within the CD81^low^CD82^low^ and CD81^high^CD82^high^ regions across the total number of CD3^+^ T, CD8^+^ T, and CD4^+^ T cells. Blue region depicts gating for low CD81 and CD82 expression, whereas the orange region signifies gating for the simultaneous high expression of CD81 and CD82. This comparison is displayed as a histogram and bar graph to demonstrate fold changes. In the bar graph, MFI values without stimulation are denoted using white bars, and MFI values after IL-2 treatment are indicated using black bars. Data is based on three independent wells. Statistical significance between groups was assessed using two-way ANOVA followed by Tukey’s multiple comparison test (*p ≤ 0.05, **p ≤ 0.01, ***p ≤ 0.001, ****p ≤ 0.0001).

Hence, we hypothesized that CD81 and CD82 might have specific characteristics related to T-cell function. After stimulation, the cells were grouped into two categories: CD81^high^CD82^high^ and CD81^low^CD82^low^ (**Figure 2A**). This sorting allowed us to investigate their impact on T cells and examine any marked trends.

We assessed the effect of activation and exhaustion markers associated with T cells. The expression levels of CD25 were 3-fold and 2.5-fold higher, respectively, in CD3^+^ and CD8^+^ T cells with CD81^high^CD82^high^ than in those with CD81^low^CD82^low^, and the expression levels of CD69 were 1.5-fold higher in CD3^+^ and CD8^+^ T cells with CD81^high^CD82^high^ than in those with CD81^low^CD82^low^. In the case of CD4^+^ T cells, despite the relatively consistent expression levels of CD81 and CD82, their impact on stimulation was less pronounced. However, there was a notable and significant difference in the expression of CD25, with a 3.5-fold higher expression in CD4^+^ T cells with CD81^high^CD82^high^ than in those with CD81^low^CD82^low^. Furthermore, CD69 expression was 1.5-fold higher under these conditions (**Figure 2B**).

In terms of exhaustion markers, we examined PD-1 and LAG-3 expression in each T-cell subset. The expression of PD-1 was 2-fold higher in CD3^+^ T cells with CD81^high^CD82^high^ than in those with CD81^low^CD82^low^, and the difference in the expression of LAG-3 was even more pronounced, with a 3-fold higher level in CD81^high^CD82^high^. In the case of CD4^+^ T cells, although the overall expression of PD-1 and LAG-3 was lower than that in other subsets, the difference in their expression was still significant. PD-1 expression was 1.5-fold higher in CD81^high^CD82^high^, and LAG-3 expression was 2-fold higher compared that in CD81^low^CD82^low^ (**Figure 2C**). In this experiment, when we analyzed the data from three different donors, the trends were similar.

Our data suggest that CD81 and CD82 are closely related to T-cell activation. Furthermore, activated T cells exhibit increased expression of CD81 and CD82. This indicated a correlation between CD81 and CD82 and the production of activation molecules.

### 3.3 Expression levels of CD81 and CD82 in T cells are sustained even after TCR stimulation and affect T-cell activation and differentiation into memory subtypes

**Figure 3:**
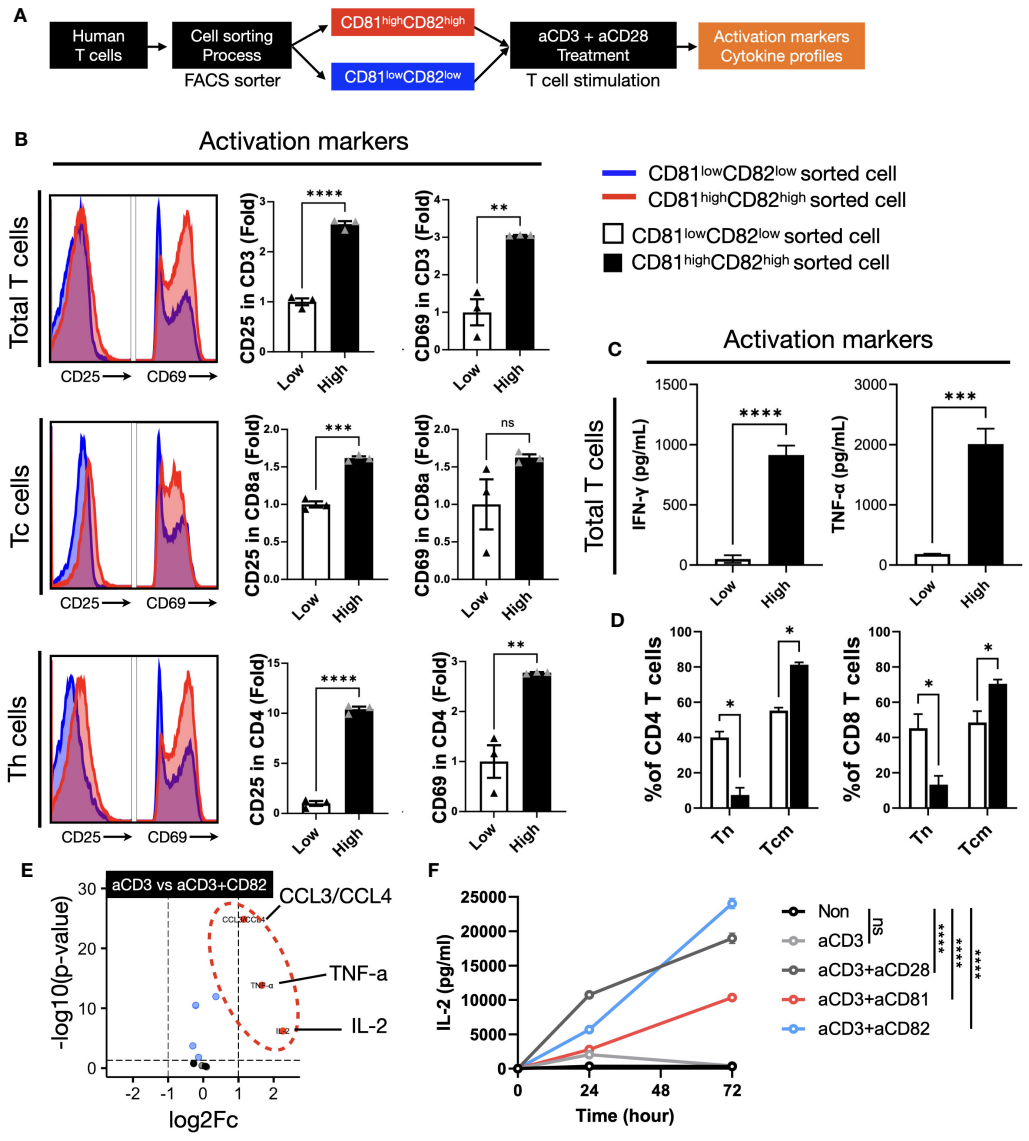
The presence of CD81 and CD82 in T cells elevates the expression of cytokines associated with TCR-related signaling. **(A)** Illustration of the experimental procedure involving IL-2 stimulation of T cells, followed by sorting into CD81^low^CD82^low^ and CD81^high^CD82^high^ T-cell subsets and subsequent stimulation with anti-CD3 antibody (0.5 μg/mL). **(B)** Evaluation of activation marker expression levels, CD25 and CD69, in various T-cell subtypes cultured using the procedure described in **(A)**. Blue histogram represents CD81^low^ and CD82^low^, and red histogram depicts CD81^high^ and CD82^high^. Bar graph illustrates the fold change in CD81^low^CD82^low^ and CD81^high^CD82^high^ populations. **(C)** Comparative analysis of the cytokine concentrations (pg/mL) of IFN-γ and TNF-α secreted by T cells cultured using the method outlined in **(A)**. **(D)** Assessment of the transition from naive T cells to central memory subtype in both CD4^+^ and CD8^+^ T cells cultured using the method described in **(A)**. This analysis explores the memory subset transition in CD81^low^CD82^low^ and CD81^high^CD82^high^ T cells after stimulation with anti-CD3 antibody (0.5 μg/mL). **(E)** Investigation of the differentiation in cytokine and chemokine secretion at the immune synapse, comparing stimulation by anti-CD3 antibody (0.5 μg/mL) with concomitant stimulation by anti-CD3 and anti-CD82 antibodies (5 μg/mL). **(F)** Comparative examination of IL-2 secretion in CD4^+^ T cells with various co-stimulatory molecules, all added at a concentration of 5 μg/mL. Statistical significance between groups was calculated using two-way ANOVA followed by Tukey’s multiple comparison test (*p ≤ 0.05, **p ≤ 0.01, ***p ≤ 0.001, ****p ≤ 0.0001). ns, not significant.

To examine the specific effects of CD81 and CD82, we sorted cells into two groups: CD81^high^CD82^high^ and CD81^low^CD82^low^. Cells were sorted after 2 days of IL-2R stimulation and anti-CD3/28 activation, as illustrated in (**Figure 3A**). Subsequently, the sorted cells were cultured on plates pre-coated with anti-CD3 (0.5 µg/ml) in medium supplemented with IL-2. This culture allowed us to assess cytokine secretion following TCR stimulation for 2 days. In the T cells before IL-2 stimulation, the expression levels of CD81 and CD82 in the CD81^low^CD82^low^ and CD81^high^CD82^high^ regions showed differences of up to 2-5 times, as illustrated in **Supplementary Figure 4A**. We assessed the expression levels of CD81 and CD82 in T cells 2 days after sorting. A 1.7-fold increase was observed in CD81 expression in Total CD3 cells compared to that in cells sorted as CD81^low^CD82^low^. In the CD8^+^ and CD4^+^ T-cell subsets, we observed increased mean fluorescence intensity values of 1.4- and 2.2-fold, respectively. Furthermore, our analysis revealed a 9-fold higher expression of CD82 in CD3^+^ T cells, 5.4-fold higher expression in CD8^+^ cells, and 9.2-fold higher expression in CD4^+^ cells than in the CD81^low^CD82^low^ subset (**Supplementary Figure 4A**).

We investigated the expression levels of CD25 and CD69 in the cells after sorting and stimulation. Our cell sorting focused on CD3^+^ T cells and we used flow cytometry to establish gates for CD8^+^ and CD4^+^ T cells, which enabled us to evaluate subtype-specific activation markers. In total, CD3^+^ T cells, the expression of CD25 in the CD81^high^CD82^high^ group was 2.5-fold higher than that in the CD81^low^CD82^low^ group, whereas that of CD69 showed a 3-fold increase. Upon examining the cell subtypes, we observed that the expression of CD25 and CD69 in CD8^+^ T cells increased 1.5-fold. In CD4^+^ T cells, the expression of CD25 was significantly higher in the CD81^high^CD82^high^ group, with a 10.4-fold difference, whereas CD69 expression was 2.7-fold higher, indicating the most prominent variation (**Figure 3B**). Notably, when we analyzed data from three different donors (n=3), the observed trends were consistent.

In T cells with increased CD81 and CD82 expression, elevated levels of CD81 and CD82 persisted even after TCR stimulation. Moreover, the expression of the activation markers, CD25 and CD69, increased significantly. These findings suggest that T cells promptly release cytokines associated with their activation upon stimulation. These immunoregulatory cytokines are closely associated with antitumor responses, leading us to explore the potential link between the upregulation of CD81 and CD82 and the production of immunoregulatory cytokines.

To assess the impact of changes in the expression levels of CD81 and CD82 on cytokine production in T cells, we cultured CD81^low^CD82^low^ and CD81^high^CD82^high^ cells separately and conducted ELISA experiments using their supernatants to measure TNF-α and IFN-γ. Notably, T cells with low CD81 and CD82 expression (CD81^low^CD82^low^) exhibited minimal secretion of cytotoxic cytokines, whereas those with high CD81 and CD82 expression (CD81^high^CD82^high^) showed a significant increase in the secretion of both cytokines. The amount of IFN-γ produced by CD81^high^CD82^high^ T cells was approximately 900 pg/mL, as compared to the control group without TCR stimulation. For TNF-α, there was a slight increase in CD81^low^CD82^low^, at around 150 pg/mL compared to the control, but this quantity was significantly lower than the 2000 pg/mL produced by CD81^high^CD82^high^.

As anticipated, the CD81^high^CD82^high^ group displayed enhanced T-cell activation with sustained CD81 and CD82 expression. Conversely, the CD81^low^CD82^low^ group showed a limited increase in cell activation, and CD81 and CD82 expression following sorting and stimulation. This suggests that low endogenous levels of CD81 and CD82 in T cells restrict their activation and responsiveness.

We hypothesized that the difference in the expression levels of CD81 and CD82 may affect the activation of T cells and variation in cytokine production, potentially influencing the proportion of memory subsets. The ratio of memory T-cell subsets is crucial for anticancer immunity, as it is associated with the expansion of antigen-specific T cells. To validate our hypothesis, under conditions similar to the previous experiment (**Figures 3B, C**), we cultured CD3^+^ T cells for 7 days and examined the changes in T-cell memory subsets through flow cytometry analysis.

In both CD4^+^ and CD8^+^ T cells, the proportion of the naive subset (CCR7^+^/CD45RA^+^) remained at approximately 40% in the CD81^low^CD82^low^ group and decreased to 7% and 10%, respectively, in the CD81^high^CD82^high^ group. In contrast, for the central memory cell subset (CCR7^+^/CD45RA^-^), in CD81^high^CD82^high^ group, there was an increase of up to 80% in CD4^+^ T cells and 70% in CD8^+^ T cells, while in the CD81^low^CD82^low^ group, CD4^+^ T cells increased by 55% and CD8^+^ T cells by 51%. No significant changes were observed in the effector memory subset (CCR7^-^/CD45RA^-^) (DATA not shown) (**Figure 3D**).

These findings imply that cells expressing high levels of CD81 and CD82 exhibit not only rapid activation but also a greater accumulation within the memory subset, potentially influencing the expansion of T cells.

### 3.4 CD81 and CD82 play a role as co-stimulatory molecules in T cells, influencing cytokine production

We observed that the expression of CD81 and CD82 on the cell surface not only affected the activation markers of T cells, but also correlated with the secretion of cytokines from T cells. CD81 and CD82 are membrane proteins expressed on T cells that function as co-stimulatory molecules. We investigated whether external stimulation of CD81 and CD82 leads to changes in cytokine expression. Reviewing previous results, while the expression patterns of CD81 and CD82 increased similarly after TCR stimulation, there was a notable difference in CD8^+^ T cells. CD81 increased by 1.5-fold, whereas CD82 increased by up to 5-fold. In CD4^+^ cells, CD82 increased over 9-fold, whereas CD81 doubled (**Supplementary Figure 4**). Additionally, we established that CD81 and CD82 increase the expression of activation markers in T cells, consequently inducing the production of cytotoxic cytokines and promoting their differentiation into central memory T-cell subsets. These findings suggest that they can serve as co-stimulatory molecules that support T-cell activation upon TCR stimulation. Therefore, to confirm the role of CD81 and CD82 as supporters of CD3 stimulation, we aimed to replicate the process of T-cell activation using conventional anti-CD28 antibodies. To mimic this, we coated plates with anti-CD3 (0.5 µg/ml) along with anti-CD81 and anti-CD82 at 5 µg/ml each and subsequently seeded the sorted cells.

The cells were cultured in pre-coated plates for 48 h and the supernatants were collected. The results indicated that there was no significant increase in cytokine levels in the group stimulated with anti-CD3/CD81 compared to the group stimulated with anti-CD3 alone (DATA not shown). However, in the anti-CD3/CD82 group, a significant increase in the production of CCL3/CCL4, TNF-α, and IL-2 was observed, with IL-2 displaying the most substantial increase (**Figure 3E**).

The production of IL-2, a crucial cytokine during the early stages of T-cell activation, plays a pivotal role in immune responses. IL-2 acts via its receptor, CD25, and is consumed within the first 48 h post-activation. To investigate the extent of IL-2 generation in both CD4^+^ and CD8^+^ T-cell subsets, these subsets were isolated from PBMC and cultured under various antibody-coating conditions, including anti-CD3 alone, anti-CD3/anti-CD28, anti-CD3/anti-CD81, or anti-CD3/anti-CD82. As a control for co-stimulation, anti-CD28 was utilized. Supernatants were collected at 24, 48, and 72-h time points from plates coated under each of these conditions, and IL-2 levels were quantified using ELISA.

In CD4^+^ T cells, IL-2 production exhibited distinct patterns under different stimulatory conditions. The anti-CD3 alone group demonstrated a peak in IL-2 production at the 24-h mark, followed by a gradual decline. In contrast, the anti-CD3/CD28 group showed an increasing trend in IL-2 production, which continued for up to 72 h, albeit with a shallower slope after the initial 24 h (**Figure 3F**). Interestingly, the anti-CD3/CD82 group exhibited sustained IL-2 production, which continued to increase with a steeper slope than that of the anti-CD3/CD28 group. While the anti-CD3/CD81 group showed lower IL-2 production than the anti-CD3/CD28 group, both groups displayed a similar increasing slope over time (**Figure 3F**).

In CD8^+^ T cells, IL-2 production reached its peak at 24 h in all groups, including anti-CD3, anti-CD3/CD28, and anti-CD3/CD82. Subsequently, IL-2 production began to decline from 48 h onwards in all groups. Notably, the anti-CD3/CD82 group demonstrated a continuous increase in IL-2 production for up to 72 h, exhibiting a more pronounced slope than the other groups (**Supplementary Figure 4B**). These findings indicate that CD82 have a substantial impact as co-stimulatory molecules on IL-2 generation, a vital T-cell growth factor, with implications for the dynamics of immune responses. In contrast, the influence of CD81 on IL-2 expression is uncertain.

### 3.5 Through bulk-RNAseq results, we demonstrated the association of CD81 and CD82 with T-cell activation and proliferation

**Figure 4:**
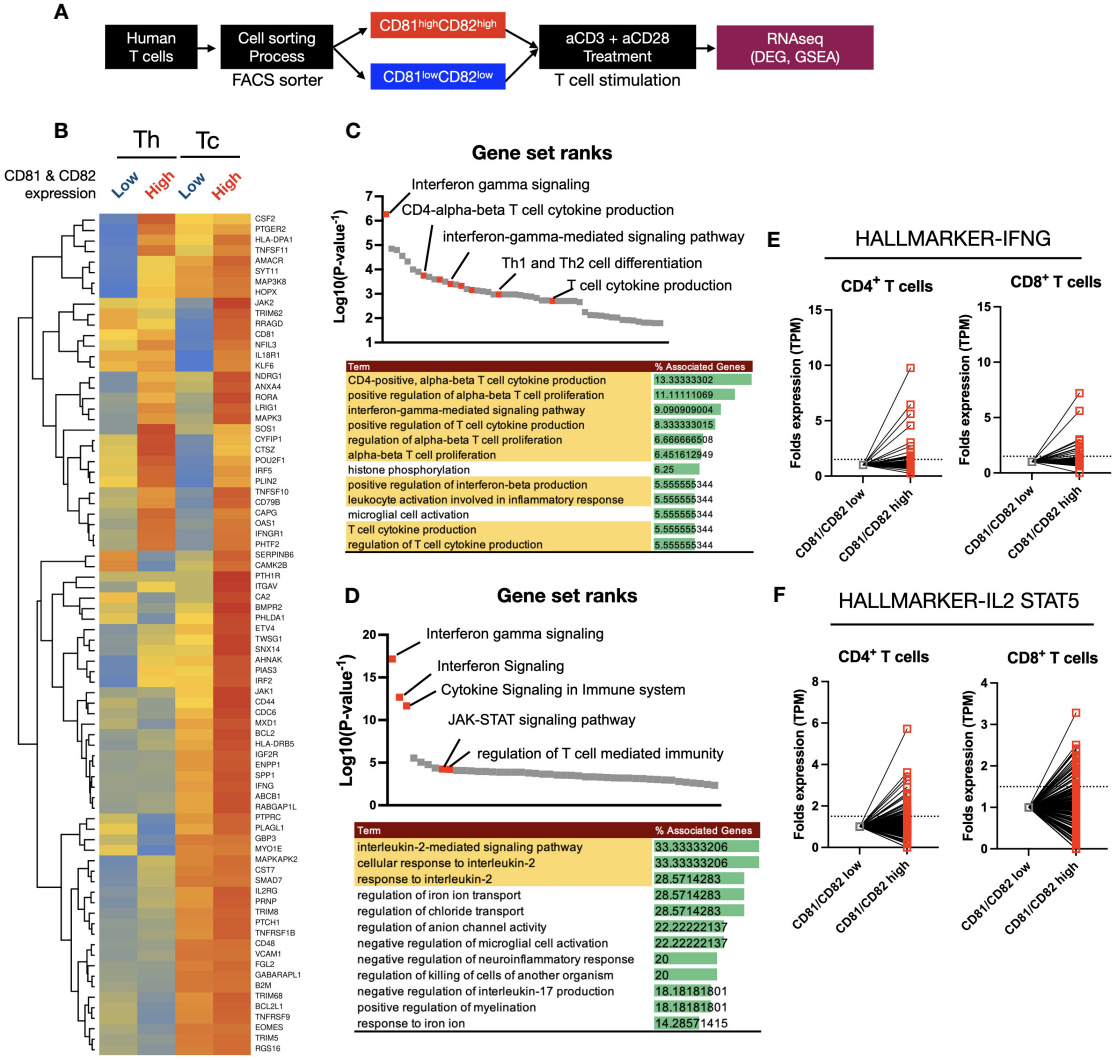
Heatmap clustering and gene set enrichment analysis (GSEA) to examine the differences and associations between T cells with high CD81 and CD82 expression and those with low CD81 and CD82 expression. **(A)** Schematic of cell sorting and RNA sequencing process. **(B)** Heatmaps showed the IFN-γ and IL-2 signaling pathway related gene set in four groups. Individual gene expression was displayed as colored boxes (red = 1, yellow = 0.5, blue = 0). **(C, D)** GSEA and KEGG pathway. Among the statistically significant gene set pathways, IFN-γ signaling, T-cell cytokine production, and IL-2-mediated signaling pathways were particularly observed. **(E)** In the CD81^high^CD82^high^ group, although the expression of each set of 198 hallmark genes related to IFN-γ and IL-2/STAT5 was similar or decreased in some areas, most areas exhibited an increase.

The results discussed in (**Figure 3**) indicate that there are differences in the expression levels of TCR-related early signaling and cytokines associated with the expression levels of CD81 and CD82. Therefore, we hypothesized that the transcriptome related to T-cell function may also undergo changes. To explore this phenomenon, we sorted the CD81^high^CD82^high^ and CD81^low^CD82^low^ groups and performed RNA sequencing (**Figure 4A**).

Using a total of 80 IFN-γ and IL-2 related gene sets obtained from KEGG Hallmark data, a heatmap clustering revealed clear differences based on CD81 and CD82 expression (**Figure 4B**). CD8^+^ T cells had higher or similar expression of all 80 genes in CD81^high^CD82^high^ than in CD81^low^CD82^low^. In contrast, CD4^+^ T cells showed higher expression of less than 32 genes in CD81^high^CD82^high^ than in CD81^low^CD82^low^. To confirm that higher expression gene sets were related to T-cell activation and proliferation pathways, we performed GSEA using the upregulated genes. First, we conducted GSEA using 32 genes that were highly expressed in both Th and Tc cells among the selected 80 genes, and ranked them accordingly. Notably, Interferon gamma signaling was ranked at the top, and pathways such as the positive regulation of alpha-beta T-cell proliferation (GO) and T-cell cytokine production (GO) were identified as related pathways (**Figure 4C**). The remaining 48 genes showed higher expression in Tc cells than in Th cells. Additionally, a trend of higher or similar expression was observed in CD81^high^CD82^high^. Therefore, we conducted GSEA using the 48 genes that showed increased expression in CD81^high^CD82^high^ Tc cells as the second set and examined their rank. Similar to that in (**Figure 4C**), the related pathways are shown in (**Figure 4D**). In particular, T-cell activation- and proliferation-related pathways, including Interferon Signaling (GO), JAK-STAT signaling pathway (GO), and IL-2 signaling pathway (GO), were identified. Based on the results of GSEA analysis, we compared the transcript per million (TPM) values of each gene in the relevant IFN-γ, IL-2/STAT5 gene sets, consisting of 198 genes each, from KEGG Hallmark as relative values (**Figures 4E, F**). Enhanced expression was observed for most genes in CD81^high^CD82^high^.

**Figure 5:**
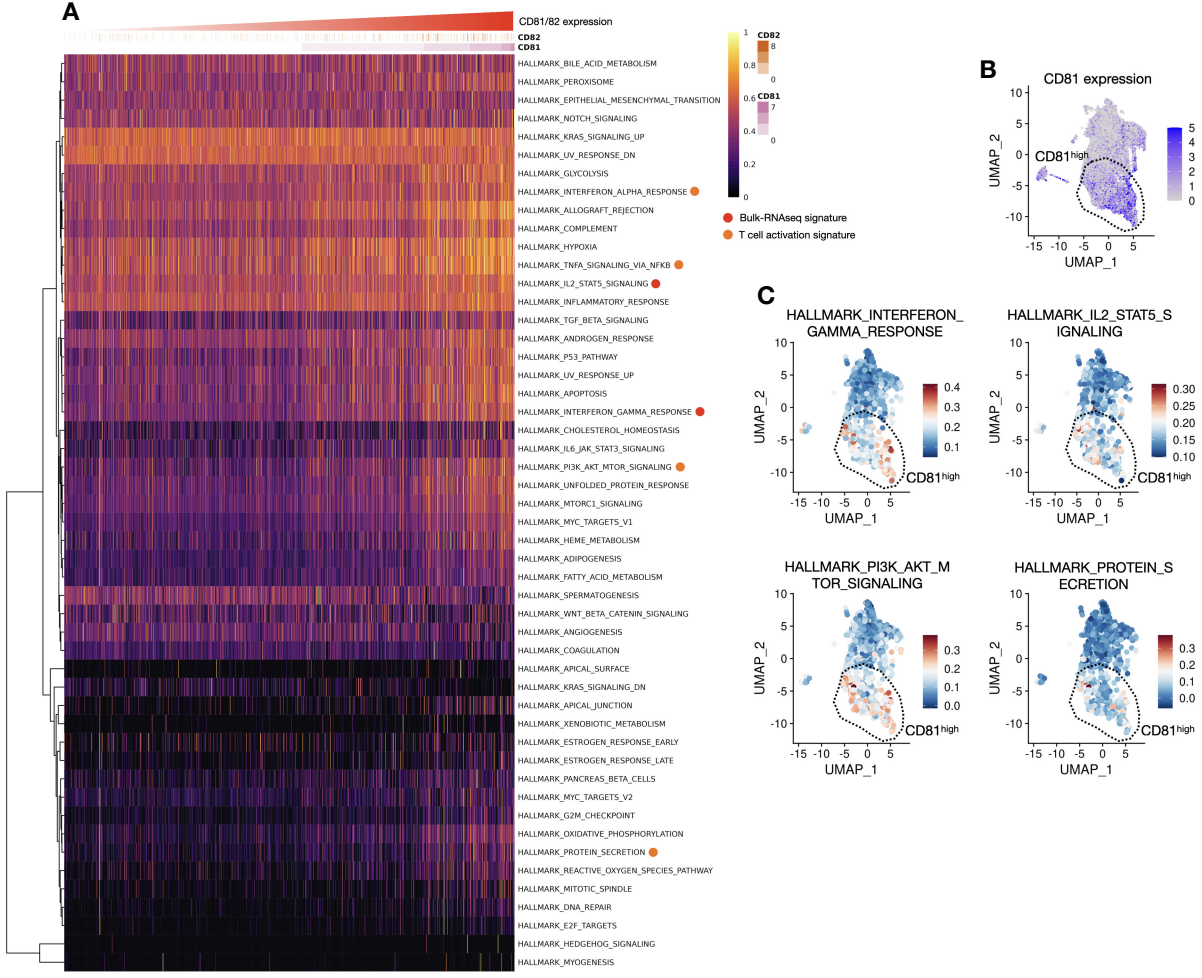
Association between CD81 and CD82 with T-cell activation and proliferation is observed in the signature gene expression analysis, aligning with the previous *in vitro* data. **(A)** Heatmap of hallmark genes serves as a visual representation of hallmark gene expression patterns, as outlined in **Supplementary Table 2**. The x-axis of this heatmap is organized in an ascending order of CD81 and CD82 expression levels, providing a clear and structured view of how these genes relate to specific cellular characteristics and functions. **(B)** Focusing on CD81^high^ region in a UMAP1-UMAP2 dimension plot, our analysis focuses on the CD81^high^ region observed within the clusters of T cells. This region, prominently distinguished by elevated CD81 expression, is highly significant in our investigation. A dashed line demarcates an enriched area, shedding light on its distinctive gene expression profile and cellular attributes. **(C)** Pathway expression insights of UMAP1-UMAP2 dimension plot unveil the expression of hallmark-related pathways. Four major categories were explored, including the IFN-γ response (upper left), IL-2 and STAT5 signaling pathways (upper right), PI3K, AKT, and mTOR signaling pathways (lower left), and protein secretion (lower right). Each of these regions is carefully separated and highlighted using dashed lines, contributing to a comprehensive understanding of the complex molecular interactions and signaling cascades at play in the cellular landscape. These enriched regions provide valuable insights into the interplay of various pathways and how they collectively influence the function and behavior of these cells.

In response to changes in T-cell population and cytokines based on the expression of CD81 and CD82, we analyzed signature gene expression, specifically related to IL-2, IFN-γ, and STAT5, in CD81^high^CD82^high^ and CD81^low^CD82^low^ T cells. The analysis was performed using samples from patients with lung adenocarcinoma, as illustrated in (**Figure 1**). We reorganized these samples based on the criteria, CD81^high^CD82^high^ and CD81^low^CD82^low^. Subsequently, we calculated the gene set enrichment scores for each cell using 50 hallmark gene sets, as determined using the SSGSEA package. When we arranged CD81 and CD82 in higher order, we observed that genes associated with IFN-γ, IL-2, and STAT5 exhibited notably higher expression in the region (indicated by the red circle). This observation strongly suggests that CD81 and CD82 are enriched in T cells within the tumor microenvironment. Moreover, the signature of T-cell activation was highly expressed (orange circle). This further underscores that elevated CD81 and CD82 levels are associated with the enhanced expression of genes linked to T-cell metabolism and proliferation (**Figure 5A**). Consequently, these genes were coexpressed in regions with high CD81 expression (**Figure 5B**).

Regarding CD82, the expression pattern was more variable and relatively low in abundance; thus, CD81 predominantly represented this phenomenon (DATA not shown). The genes associated with IFN-γ, IL-2, and STAT5 signaling, which were highly expressed in the CD81-high region, were enriched in the same spatial location. Additionally, the PI3K, AKT, and mTOR signatures were highly expressed in the same area. Notably, protein secretion, indicative of cytokine release, was elevated (**Figure 5C**). The *in silico* analysis results are consistent with our *in vitro* findings, confirming a consistent trend in the expression of signature genes in T cells within the tumor microenvironment.

### 3.6 CD81 and CD82 overexpression in T cells induces cytolysis, suggesting CD81 and CD82 as promising targets for immunotherapy

The activation of T cells and subsequent expression of cytokines play crucial roles in inducing cytolysis in cancer cells. To explore the impact of T-cell activation and cytokine expression on cancer cell cytolysis, particularly that influenced by CD81 and CD82, as observed in our preceding results, we embarked on a series of investigations.

**Figure 6:**
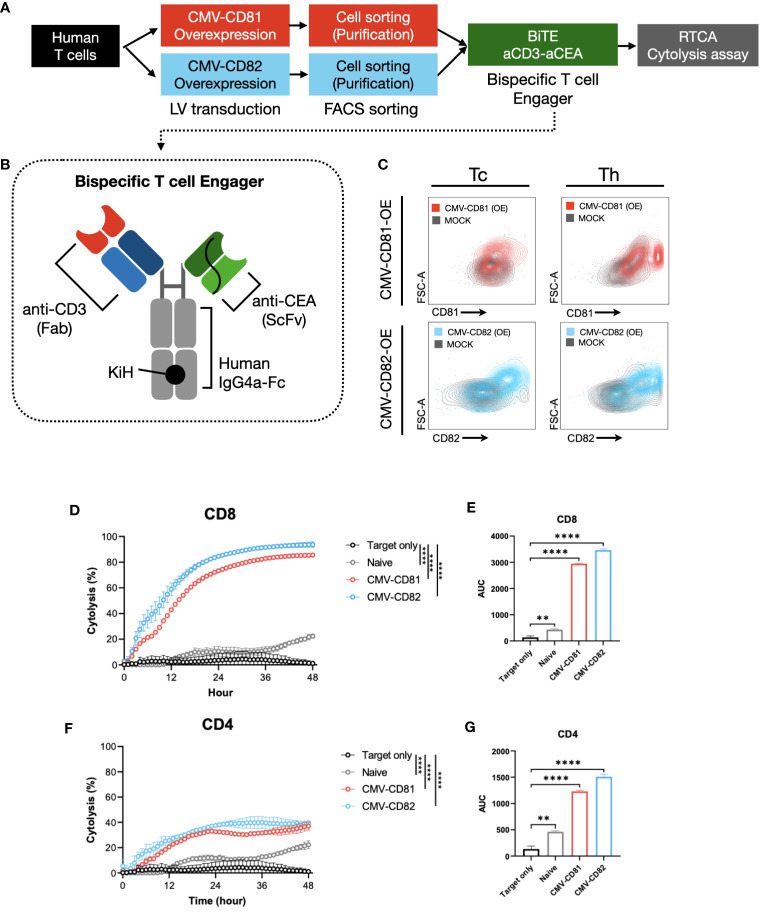
Increased levels of CD81 and CD82 expression within T-cell clusters boost their cytotoxic potential. **(A)** Overview of the experimental method and materials used. **(B)** Schematic of the bispecific T-cell engager, incorporating binding regions for CD3 and CEA. **(C)** Flow cytometry to assess transduction efficiency. Cells were separated into CD4^+^ and CD8^+^ T-cell subsets. Gray graph represents the baseline expression level, red graph illustrates the expression of CD81 after transduction with CMV-CD81, and blue graph shows the expression of CD82 after transduction with CMV-CD82. Data was analyzed 3 days after transduction. **(D)** Cytolysis graph comparing naive CD8^+^ T cells with CD81 and CD82 overexpression. Statistical significance was calculated at 48 h. The Effector : Target (E:T) ratio is 5:1, and target cells were seeded at 1E4, with bispecific engager added at 25 nM. **(E)** Statistical significance calculated for the area under the curve of the cytolysis data presented in **(D)**. **(F)** Cytolysis graph comparing naive CD4^+^ T cells with CD81 and CD82 overexpression. Statistical significance was calculated at 48 h. The E:T ratio is 5:1, and target cells were seeded at 1E4, with bispecific engager added at 25 nM. **(G)** Statistical significance calculated for the area under the curve of the cytolysis data presented in **(F)**. Statistical significance between groups was calculated using two-way ANOVA followed by Tukey’s multiple comparison test (**p ≤ 0.01, ****p ≤ 0.0001).

Our strategy involved the isolation of CD4+ and CD8+ T cells from PBMCs, followed by the overexpression of CD81 and CD82 in these cells. (**Figure 6A**). The extent of overexpression in each T-cell subtype was verified using flow cytometry (**Figure 6C**). Subsequently, we performed co-culture experiments involving the target cells, including CEA-overexpressing CHO-K1 cells, and continuously monitored the real-time cytolysis of these targets, using a real-time cell analysis (RTCA) system. To ensure effective recognition of the target cell antigen during co-culture, we introduced a bispecific antibody capable of simultaneously recognizing CEA (a specific antigen expressed on target cells) and CD3 (expressed on T cells) as an engager (**Figure 6B**).

CD8^+^ T cells overexpressing CD81 and CD82 exhibit a remarkable real-time cytolytic effect when co-cultured with target cells. This resulted in up to 80% of the target cells undergoing cytolysis at the 36-hour mark (**Figures 6D, E**). Furthermore, co-culture of CD4^+^ T cells with the target cells yielded a markedly result, with approximately 40% of the target cells undergoing cytolysis at the 36-hour mark (**Figures 6F, G**).

Thus, the expression of CD81 and CD82 not only plays a role in influencing cytokine production in both CD8^+^ and CD4^+^ T cells but also exerts a substantial influence on the cytolysis of target cells expressing specific antigens. A notable aspect of these findings was the absence of significant differences between the CD81 and CD82 groups, highlighting the potential of CD81 and CD82 as crucial contributors to the induction of cytolysis in target cells. These findings suggest that CD81 and CD82 are promising candidates for immunotherapy.

## 4 Discussion

Tetraspanin-induced membrane protein organization has been proposed as a key mechanism that regulates immune receptor function (20, 21). Therefore, comprehensive research into their precise roles in anticancer immunity is of importance. Apart from their involvement in TCR signaling, tetraspanins, such as CD81 and CD82, which are expressed in cancer cells, also influence the promotion or inhibition of tumor metastasis (22, 23). Hence, from the perspective of studying anticancer immunity, it is important to differentiate between the roles of tetraspanins in T cells and tumor cells. With the advancement of single-cell RNA technology, it has become possible not only to distinguish cells within tumors but also to identify the expression levels of surface markers on each cell. Therefore, we were able to conduct a study using single-cell RNA data from non-small cell lung cancer patients to differentiate tetraspanins associated with the activation of T cells within the TME. To delineate the roles of tetraspanins in each cell type, we analyzed data obtained using single-cell RNA sequencing technology from tissue samples of 19 patients with stages 1-2. In total, 173, 023 cells were analyzed, and among the 33 genes belonging to the human tetraspanin family, eight tetraspanins were identified in regions with high TPS scores. Among these eight tetraspanins, CD81 and CD82 showed the highest expression in areas with high TPS in the TME, particularly in the T-cell lineage (**Figure 1G**). Investigation into the cell types exhibiting this increased expression revealed not only cytotoxic T cells but also various other T-cell types demonstrating high levels of expression (**Figure 1H**). Additionally, T cells expressing CD81 and CD82 showed high expression of genes associated with activation. Considering that conditions in the TME are hostile to T cells, the strong correlation between activated T cells in the TME and the tetraspanins CD81 and CD82 is intriguing.

The functions of proteins belonging to the tetraspanin family, CD81 and CD82, have been studied in various cells since their discovery in 1988 and 1991, respectively (24). Particularly in the field of anticancer immunity, research on CD81 gained traction after a 2012 study reported that knocking out CD81 resulted in the regulation of TCR signaling (25). Subsequent studies have confirmed the involvement of CD81 in TCR signal transduction (26). While it is clear that CD81 plays a role in TCR signaling, there are cases where the findings are contradictory and the exact mechanism is yet to be elucidated. Studies suggest that CD82 acts as an inhibitor in solid tumors (27), and research has indicated its function as a co-stimulatory molecule. However, a clear mechanism related to the TCR has not yet been established. Particularly, in the field of anticancer immunity, questions regarding both CD81 and CD82 remain unanswered.

There is substantial evidence suggesting the involvement of CD81 and CD82 in TCR signaling (28, 29). This could potentially lead to the transcription of genes involved in the activation and proliferation of T cells, such as IL-2, after the activation of the downstream signal ZAP70 following TCR stimulation (30). To confirm the roles of CD81 and CD82 in T cells, as observed in the single-cell analysis, we conducted *in vitro* experiments using IL-2-stimulated T cells. We found that the distributions of CD81 and CD82 changed over time in IL-2-activated T cells (**Supplementary Figure 3**). Tetraspanins, such as CD9, CD53, CD81, and CD82, have been suggested to be co-stimulatory molecules for T cells (31). However, in certain cases, a mouse knockout model for CD81 *in vitro* showed T-cell over-proliferation (32), indicating a negative regulatory function of CD81. To confirm these possibilities, we further divided IL-2-stimulated T cells into regions with high and low expression of CD81 and CD82 and analyzed them separately as CD81^high^CD82^high^ and CD81^low^CD82^low^ (**Figure 2A**). The difference in expression levels of CD25 and CD69, activation markers for T cells, between CD81^high^CD82^high^ and CD81^low^CD82^low^ regions was clear. These results demonstrate a clear correlation between CD81 and CD82 and T-cell activation. Additionally, these cells exhibited notable differences in the exhaustion markers PD-1 and LAG-3, which inevitably followed T-cell activation. The expression levels of these fatigue markers, as observed in CD81 knockouts (32), could potentially influence the activation of T cells under experimental conditions.

We confirmed that T cells sorted from the CD81^high^CD82^high^ and CD81^low^CD82^low^ regions maintained differences in activation markers, even when reactivated with antiCD3 (**Figure 3**). Furthermore, ELISA revealed the distinct expression levels of the key cytokines IFN-γ and TNF-α, which play a crucial role in anticancer action. After 7 days of culture, T cells in the CD81^high^CD82^high^ state were found to promote Tcm differentiation. These results align with those of previous studies, suggesting the potential utilization of CD81 in CAR-T cell development (33). Furthermore, we confirmed that T cells in the CD81^high^CD82^high^ state are the primary cells expressing anticancer-related cytokines IFN-γ and TNF-α (**Figure 3C**).

These effects were also observed in externally stimulated CD81 and CD82 + cells (**Figures 3E, F**). Some studies have suggested that CD81 and CD82 act as co-stimulatory molecules in the mouse spleen and Jurkat cells (34, 35). In our experiments with human PBMCs, we confirmed that CD82 can produce IL-2. In our study, CD82 produced as much IL-2 in CD4 and CD8 cells as CD28; it is worth noting that CD82 enhanced IL-2 production more significantly than CD28 co-stimulation, which is known to increase IL-2 production by 30–100 fold (36). Although CD81 did not increase IL-2 production as much as CD28, it sustained the production of IL-2 in CD4 cells. Although the cytokine mechanisms of T cells in the CD81^high^CD82^high^ state and externally stimulated CD81 and CD82 may differ, it is important to note that IL-2 production within the TME is crucial for the initial activation of T cells because Tregs in the TME rapidly consume IL-2 to regulate T-cell immunity (37). Given the high expression of CD25 in CD81^high^CD82^high^ T cells, it is intriguing that despite the activation of the IL-2 receptor CD25, IL-2 secretion is sustained.

We also conducted bulk RNA sequencing to identify the origin of effects of CD81 and CD82 induced T cells. Based on scRNAseq. Of T cells, we analyzed the sorted T cells from the CD81^high^CD82^high^ and CD81^low^CD82^low^ regions. When analyzing sets of genes related to IL-2 signaling and IFN-γ production, we detected a clear difference based on the expression of CD81 and CD82, with 32 gene sets being observed for CD4 cells and 80 gene sets for CD8 cells (**Figure 4**). In particular, the prominent difference in IFN-γ and IL-2-STAT5 gene sets indicates that T cells in the CD81^high^CD82^high^ state are associated with the stabilization and expression of mRNA, which in turn are related to activation-related cytokines. To confirm the results of bulk RNA sequencing *in vitro*, we performed single-cell analysis. When sorting cells from high CD81 and CD82 expression to low expression, we observed a distinct pattern of gene expression related to IFN-γ and IL-2-STAT5, which not only showed clear differences in the bulk RNA analysis but also spatially aligned with the regions (**Figure 5**). IL-2-STAT5 signaling not only regulates the activation of CD4 effector T cells in various aspects of T-cell immunity, but also exerts diverse effects on the activation of Tregs and CD8 cells (38, 39). Despite contrasting results regarding the influence of IL-2-STAT5 on the TME (40), studies indicating the role played by IL-2-STAT5 signaling in maintaining the response of CD8 cells and cytokines (such as IL-7 and IL-15) remain valid. Furthermore, IL-2 is crucial for the regulation and equilibrium of CD8 cells (41–43).

We confirmed an increase in cytotoxicity against target cancer cells when CD81 and CD82 were overexpressed in the T cells. These results indicate that the mere expression of CD81 and CD82 activates T-cell functions (**Figure 6**). These effects were likely associated with increased cytokine levels in both CD4 and CD8 cells. Although cytotoxicity against target cells did not completely disappear when CD81 and CD82 were knocked out (data not shown), this limitation may stem from the possibility that the 33 identified tetraspanins accumulate and act in the immunological synapses of tetraspanin-enriched microdomains (TEMs), thereby either sharing overlapping functions or bridging with each other and giving rise to unique characteristics (24, 44).

Identifying factors that regulate the activity of TILs in a hostile TME is crucial for tumor immunology research (45, 46). This study is the first to elucidate the role of tetraspanins in TILs within the TME of patients with non-small cell carcinoma, following the observation of CD81 expression in the innate immune landscape of lung adenocarcinoma (47).

**Figure 7:**
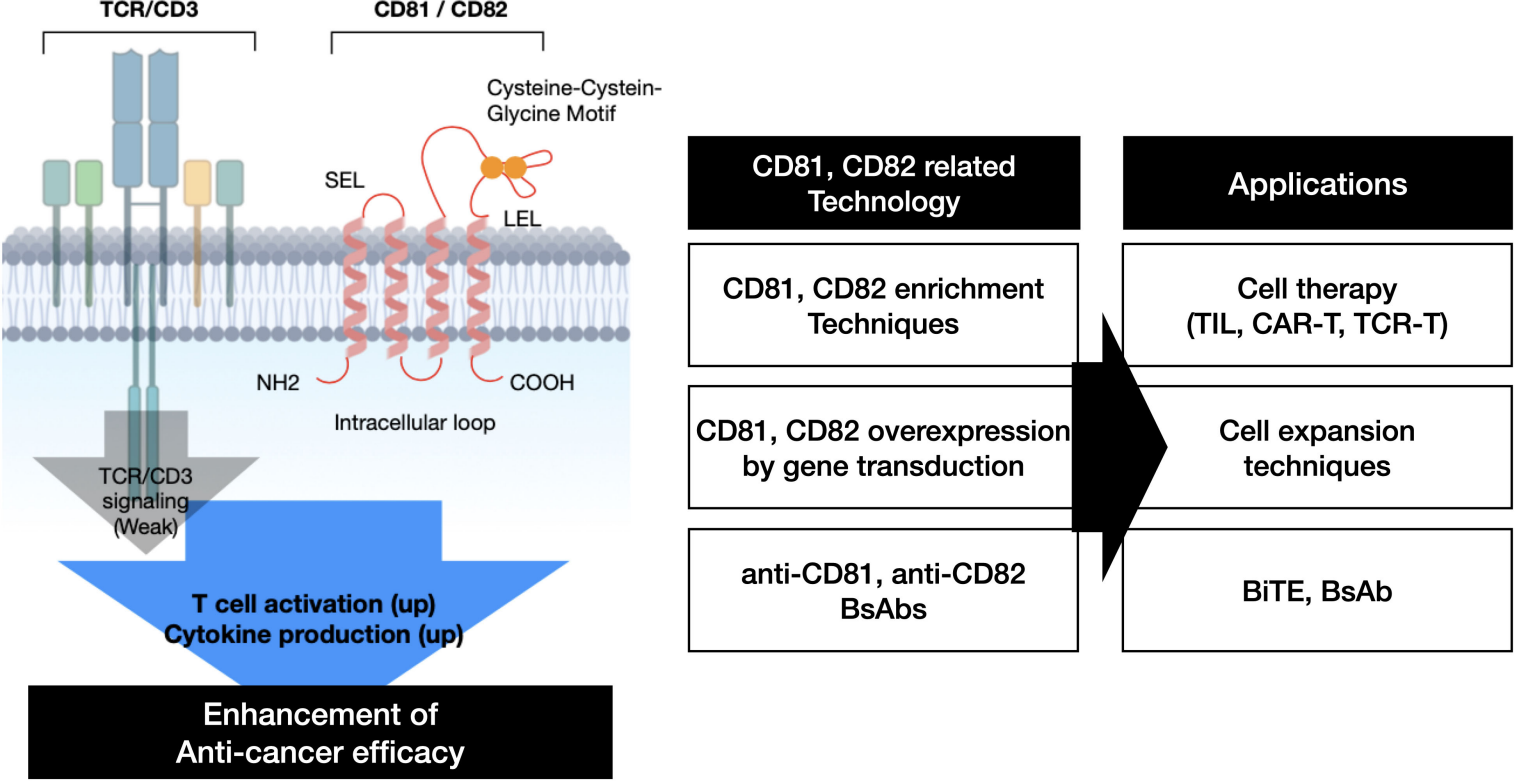
Schematic representation of the summary.

The findings summarized in this research article are presented in (**Figure 7**). CD81 and CD82 have demonstrated their potential as co-stimulatory molecules that can bolster TCR signaling in T cells, thereby augmenting T cell activation and cytokine production. We substantiated these capabilities through an analysis of their expression levels following IL-2R stimulation, assessment of cytokine production in response to their stimulation, gene overexpression in T cells, and subsequent evaluation of cytolysis using a bispecific antibody. These results suggest that CD81 and CD82, much like co-stimulatory molecules in T cells, inherently possess the ability to precisely regulate T cell activation based on their expression and stimulation levels. Therefore, utilizing CD81 and CD82 as co-stimulatory molecules in the development of Bispecific T cell Engager (BiTE), CAR-T and TCR-T could potentially lead to the development of novel anticancer therapies.

In conclusion, we found that CD81 and CD82 serve as specific markers of activated T cells within the TME. CD81 and CD82 are correlated with the activation and regulation of T cells and may also mediate the regulation of relevant cytokines. Overall, our findings provide insights into the molecular mechanisms regulating TILs present in the TME of NSCLC and suggest that CD81 and CD82 be used as associated markers. These findings may serve as a foundation for the development of new immunotherapies targeting TMEs.

Our research has a few limitations. We have not yet clearly distinguished the precise roles of CD81 and CD82. According to *in vitro* experiment results, the expression of CD82 significantly increases in activated T cells. Additionally, during stimulation, CD82 shows a more pronounced production of cytokines such as IL-2 and IFN-g compared to CD81. However, since CD81 also exhibits a similar trend, there may be some overlap in their actions. Furthermore, it has been observed that both CD81 and CD82 influence cytokine production in CD4 cells and affect cytotoxicity against cancer cells in CD8 cells. However, there is a need to differentiate the mechanisms in these two different types. Previous research indicates that both CD81 and CD82 contribute to the formation of the immune synapse in T cells, but they play distinct roles (26, 48). CD81 acts as a crucial regulator in the central SMAC (cSMAC) by binding with CD3ζ, controlling the maturation stages of the immunological synapse (IS) through interactions with CD3 and ICAM-1. On the other hand, CD82 accumulates in the peripheral SMAC (pSMAC), triggering actin polymerization and activation of the Rho GTPase pathway. This pathway activation enhances the phosphorylation of TCR signaling molecules LAT and ZAP-70. Combining these previous findings with our results, it appears that further single-cell analysis related to TCR synapse is needed to distinguish the roles of these two tetraspanins more clearly. Additionally, this should help elucidating the distinct roles of CD81 and CD82 in CD4 and CD8 cells.

## Data availability statement

The original codes presented in the study are publicly available. This data can be found here: https://github.com/DongKwonKIM/CD81_CD82. The datasets presented in this study can be found in online repositories. The names of the repository/repositories and accession number(s) can be found below: GSE252148 (GEO).

## Ethics statement

The study protocol was approved by the Institutional Review Board of Severance Hospital (IRB) under study numbers 4-2016-0788 and 4-87 2014-0775. Written informed consent for tissue and clinical information was obtained from all patients.

## Author contributions

KN: Writing – original draft, Conceptualization, Data curation, Investigation. SL: Writing – original draft, Conceptualization, Data curation, Investigation. DK: Writing – original draft, Data curation, Formal analysis, Software. YSK: Writing – original draft, Investigation. JH: Writing – original draft. S-SK: Writing – original draft. SB: Writing – original draft. CL: Writing – original draft. SY: Writing – original draft. YH: Writing – original draft. MK: Writing – original draft. HH: Writing – original draft. YTK: Writing – original draft. JK: Writing – original draft. SJ: Writing – original draft. YB: Writing – original draft. JL: Writing – original draft. SML: Writing – original draft. MH: Writing – original draft. K-HP: Writing – original draft, Conceptualization, Data curation, Methodology, Project administration, Writing – review & editing, Formal analysis, Supervision. BC: Conceptualization, Data curation, Funding acquisition, Methodology, Project administration, Writing – original draft, Writing – review & editing.
