# Supplementary material for: CD81 and CD82 expressing tumor-infiltrating lymphocytes in the NSCLC tumor microenvironment play a crucial role in T-cell activation and cytokine production
Source: Front Immunol. 2024 Mar 7;15:1336246. doi: 10.3389/fimmu.2024.1336246 (PMC10954780; doi:10.3389/fimmu.2024.1336246)
Supplement: Supplementary file 1 [file DataSheet_1.pdf]

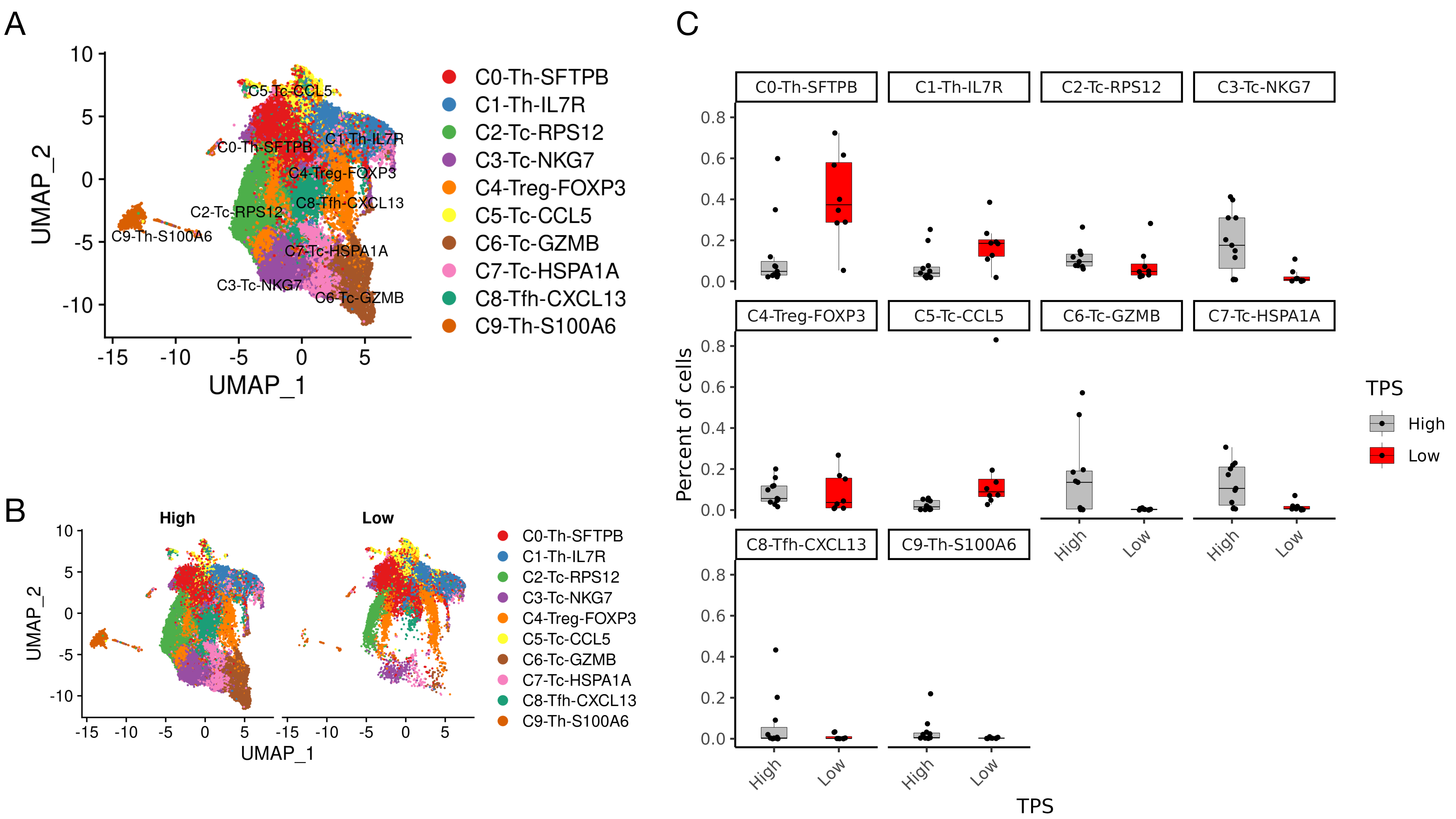

Supplementary Figure 1. (A) Cells are annotated on the UMAP1-UMAP2 Dimension Plot, highlighting distinct cell types: helper T cells, cytotoxic T cells, regulatory T cells, and follicular helper T cells. The cells are categorized into 10 clusters. (B) Shown in this panel is a UMAP1-UMAP2 Dimension Plot illustrating the enrichment of annotated cells in TPS categories, distinguishing TPS low and TPS high cells. (C) Bar plots depict the distribution of cells within each T cell cluster, differentiating between TPS high and TPS low cells. The values are expressed as percentages of the total cells in each cluster.

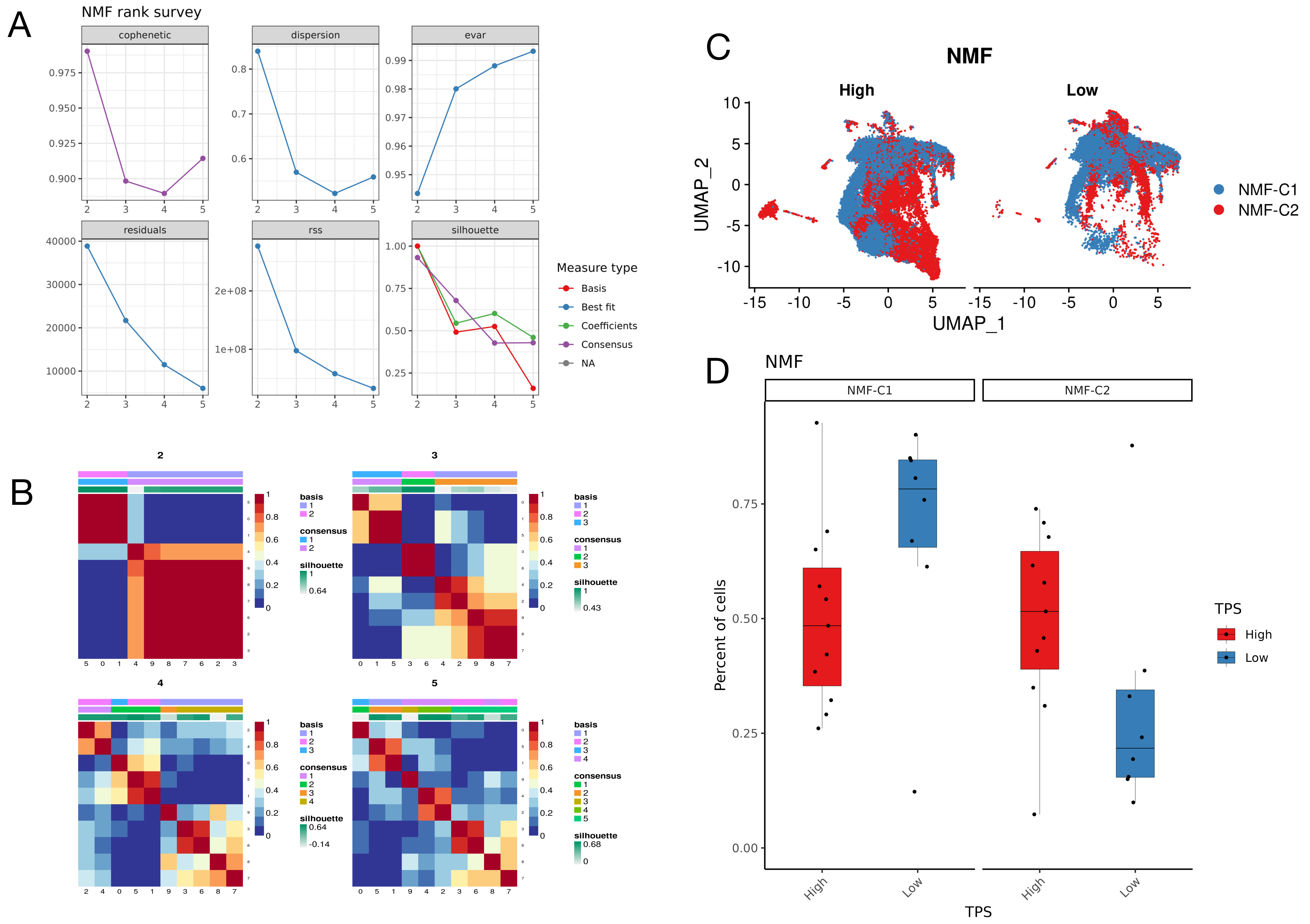

Supplementary Figure 2. (A) Cophenetic correlation coefficients associated with different numbers of clusters. The y-axis represents the cophenetic correlation coefficient, and the x-axis represents the number of clusters. The arrow displays a specific point or value. (B) Heat map of the reordered consensus matrix. In the heat map, dark blue indicates samples that were never assigned to the same cluster, while red indicates samples that were consistently assigned to the same cluster. (C) This panel illustrates NMF clustering into 2 distinct clusters represented in the UMAP1-UMAP2 Dimension plot. The position of the dot on the plot signifies the TPS score, with the left side indicating high scores and the right side indicating low scores. (D) Bar plot provides an in-depth view of the correlation between the TPS score and the two NMF clusters (Cluster 1 and Cluster 2). The correlation values are expressed as a percentage of the total cells analyzed.

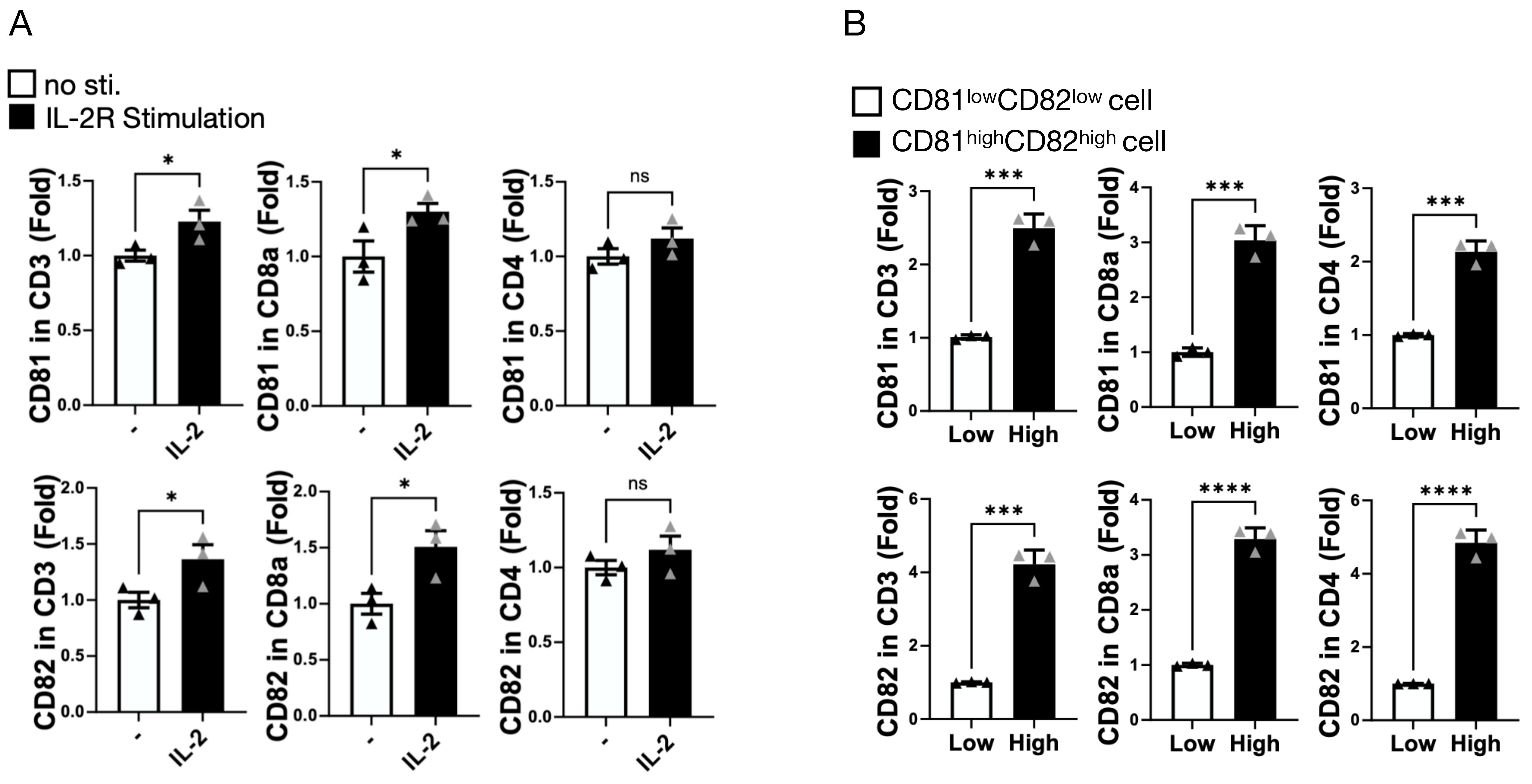

Supplementary Figure 3. (A) Flow cytometry analysis displays the fold change in the expression levels of CD81 and CD82 in in various T cell subtypes following treatment with IL-2 (50 U/ml). The white bar represents the baseline expression on Day 0, without IL-2, while the black bar illustrates the expression on Day 2 after IL-2 treatment. (B)Flow cytometry analysis displays the fold change in the expression levels of CD81 and CD82 in in various T cell subtypes before stimulation with IL-2. The data is obtained from three independent wells. Statistical significance between groups was assessed using a two-way ANOVA followed by Tukey's multiple comparisons tests (*p≤0.05, **p≤0.01, ***p≤0.001, ****p≤0.0001).

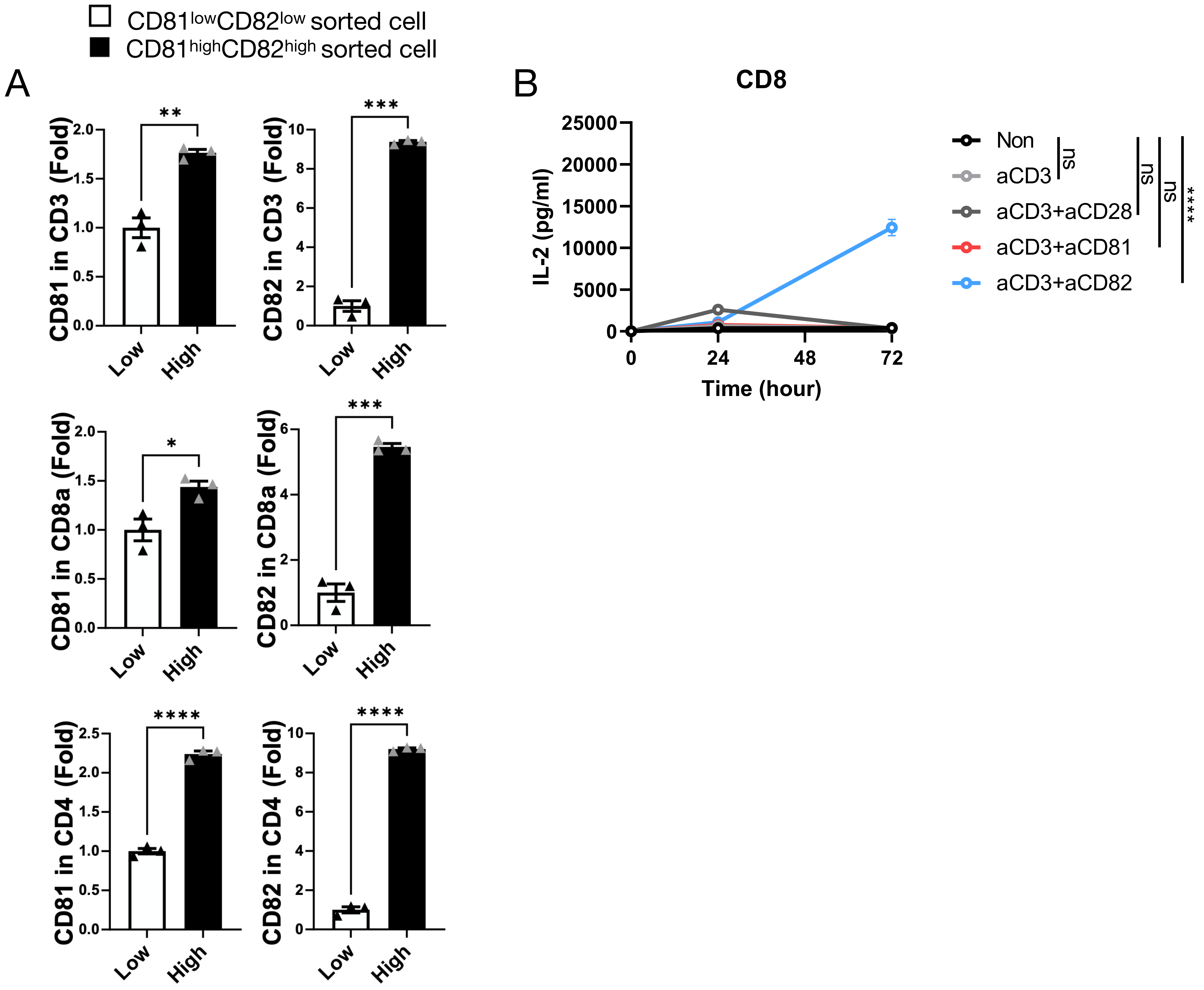

Supplementary Figure 4. (A) Assessment of the fold change in the expression levels of CD81 and CD82 in different T cell subtypes after sorting. (B) Comparative analysis of IL-2 secretion in $CD8^{+}$ T cells with various co-stimulatory molecules, all added at a concentration of 5 μg/ml. Statistical significance between groups was determined using a two-way ANOVA followed by Tukey's multiple comparisons tests (*p≤0.05, **p≤0.01, ***p≤0.001, ****p≤0.0001).

Supplementary Table 1. Demographic and clinical information from the patient samples with NSCLC, including age, sex, cancer grade, cancer stage, and metastasis.

	Total Number (n)	Percentage (%)
**Age**		
11-20	-	-
21-30	-	-
31-40	-	-
41-50	-	-
51-60	3	15.8
61-70	12	63.2
71-80	4	21.1
81-90	-	-
**Sex**		
Female	3	15.8
Male	16	84.2
**Stage**		
Unknown	1	5.3
I	6	31.6
II	3	15.8
III	7	36.8
IV	2	10.5
**Type**		
WT	18	94.7
Normal	1	5.3
**Metastasis**		
Metastasis	2	10.5
None	17	89.5

Supplementary Table 2. Top 15 genes in several cell types in tumor microenvironment. Red circle indicates T cell subset.

● T cell subsets

Rank	Adventitial Fibroblast	Airway Smooth Muscle	Alveolar Epithelial Type 1	Alveolar Epithelial Type 2	Alveolar Fibroblast	Artery	B	Basal	Basophil/Mast 1	Basophil/Mast 2	Bronchial Vessel 1	Bronchial Vessel 2	Capillary	Capillary Aerocyte
1	DCN	TAGLN	EMP2	NAPSA	MGP	CLEC3B	CD79A	KRT19	TPSAB1	TPSAB1	SPARCL1	SPARCL1	IFI27	RAMP2
2	MGP	MYL9	GPRC5A	SFTPB	DCN	CLDN5	MS4A1	KRT8	TPSB2	CPA3	IGFBP7	SOCS3	EPAS1	HPGD
3	FBLN1	ACTA2	KRT7	SLC34A2	LUM	MGP	CD37	CLDN4	CPA3	TPSB2	VWF	CLEC14A	RAMP2	CLDN5
4	SPARCL1	IGFBP7	LMO7	WFDC2	PTGDS	VWF	HLA-DRA	KRT18	TPSD1	TPSD1	A2M	IGFBP7	CLEC3B	SPARC
5	LUM	TPM2	KRT18	MUC1	A2M	A2M	CD74	TACSTD2	GATA2	KIT	PECAM1	RAMP2	EGFL7	SPARCL1
6	COL1A2	MYH11	KRT19	NPC2	MFAP4	CLEC14A	BANK1	S100A6	LTC4S	GATA2	RAMP2	TM4SF1	CLDN5	IFI27
7	SERPING1	ADIRF	CAV1	SFTA2	FBLN1	SPARCL1	HLA-DPB1	WFDC2	SLC18A2	SLC18A2	IGFBP4	EGR1	TIMP3	PECAM1
8	MMP2	CALD1	VEGFA	S100A6	RARRES2	EPAS1	HLA-DPA1	SFN	KIT	HDC	MGP	CAV1	AQP1	CAV1
9	SERPINF1	SPARCL1	CD55	KRT19	ADH1B	RAMP2	LTB	KRT7	MS4A2	IL1RL1	GNG11	A2M	CAV1	APP
10	C1S	MGP	CCN1	CLDN4	COL1A2	IFI27	LAPTM5	SPINT2	HDC	NFKBIZ	PLVAP	GNG11	SPARCL1	AQP1
11	ADH1B	TPM1	KRT8	KRT8	SPARCL1	IGFBP7	CD83	PERP	HPGDS	CD69	HSPG2	PECAM1	PECAM1	EGFL7
12	MFAP4	BGN	AGER	SERPINA1	C1S	ENPP2	CD19	S100A11	VWA5A	CLU	AQP1	CLDN5	TM4SF1	GNG11
13	IGFBP4	PPP1R14A	RAB11FIP1	SFTPA2	BGN	PECAM1	HLA-DQA1	IFITM3	CLU	HPGDS	SPARC	ZFP36	SPARC	EDNRB
14	NBL1	SOD3	HOPX	EPCAM	NBL1	ID3	HLA-DQB1	HSPB1	RHEX	LTC4S	CRIP2	IGFBP4	GNG11	ESAM
15	C3	MUSTN1	TACSTD2	HOPX	COL6A2	PTPRB	HLA-DRB1	APP	IL1RL1	MS4A2	CLEC14A	VWF	RNASE1	TIMP3

Rank	Capillary Intermediate 1	Capillary Intermediate 2	CD4+ Memory/Effector T ●	CD4+ Naive T ●	CD8+ Memory/Effector T ●	CD8+ Naive T ●	Ciliated	Classical Monocyte	Club	Differentiating Basal	EREG+ Dendritic	Fibromyocyte	Goblet	IGSF21+ Dendritic
1	AL031658.1	CLDN5	IL7R	LTB	CCL5	CCL5	CAPS	S100A8	SFTPB	KRT19	IFI30	CLU	SLPI	HLA-DRA
2	KRT222	SPARCL1	CD3E	SARAF	NKG7	NKG7	C20orf85	S100A9	WFDC2	WFDC2	PLAUR	TAGLN	KRT19	CD74
3	LINC01082	RAMP2	IL32	IL7R	CST7	CD3E	TPPP3	LYZ	CYB5A	KRT7	TYROBP	IGFBP7	LCN2	C1QC
4	DHH	TIMP3	SPOCK2	CD3E	GZMK	CD8A	C9orf24	FCN1	SLC34A2	KRT17	FCER1G	MYH11	FXYD3	HLA-DPA1
5	FOXF1	SPARC	CD3D	EEF1A1	GZMA	CD3D	TSPAN1	IFI30	CLDN4	KRT8	LYZ	MFAP4	GSTP1	HLA-DRB1
6	LINC02344	IFI27	LTB	CCR7	CD8A	IL32	RSPH1	TYROBP	MUC1	CLDN4	CST3	MYL9	AGR2	HLA-DPB1
7	APLNR	EGFL7	CXCR4	SPOCK2	CD3E	GZMA	C5orf49	VCAN	KRT18	KRT18	HLA-DRA	ACTA2	TACSTD2	C1QA
8	CYYR1	AQP1	TNFAIP3	RPS12	CD3D	CST7	IGFBP7	AIF1	SLPI	SPINT2	AIF1	MGP	ELF3	TYROBP
9	AC009065.8	CLEC3B	CD2	RPL13	IL32	GZMB	PRDX5	FCER1G	KRT8	TACSTD2	HLA-DRB1	A2M	KRT8	IFI30
10	GRPEL2	EPAS1	CD5	IL32	DUSP2	CD7	FAM183A	CST3	CXCL17	S100A6	HLA-DPB1	CALD1	SPINT2	CST3
11	ZNF467	GNG11	LCK	RPL32	CXCR4	CXCR4	SNTN	PLAUR	KRT19	MDK	HLA-DPA1	TPM2	CLDN7	HLA-DQB1
12	PODXL	PECAM1	ETS1	RPL3	CD8B	GZMH	PIFO	SLC11A1	HOPX	IFITM3	CD74	SPARCL1	MUC1	FCER1G
13	AC105101.2	CAV1	ZFP36L2	RPS27A	CCL4	CTSW	ODF3B	LST1	PIGR	ELF3	SPI1	SELENOM	CLDN4	HLA-DRB5
14	HHEX	SPTBN1	CD6	FXYD5	GZMH	PRF1	C11orf88	CD14	MGST1	TM4SF1	FTH1	AEBP1	KRT7	C1QB
15	GIMAP6	A2M	ICOS	RPL11	HCST	ZFP36L2	C9orf116	TYMP	NAPSA	ANXA2	PSAP	SERPING1	KRT18	CD14

Rank	Intermediate Monocyte	Ionocyte	Lipofibroblast	Lymphatic	Macrophage	Mesothelial	Mucous	Myeloid Dendritic Type 1	Myeloid Dendritic Type 2	Myofibroblast	Natural Killer	Natural Killer T	Neuroendocrine	Nonclassical Monocyte
1	AIF1	KRT19	COL1A2	CCL21	IFI30	SPARC	SLPI	HLA-DPB1	HLA-DPB1	COL1A2	GNLY	GNLY	EPHX1	LST1
2	IFI30	KRT8	COL6A2	IGFBP7	C1QA	TIMP1	BPIFB1	CD74	HLA-DPA1	COL3A1	NKG7	CCL5	PDLIM5	AIF1
3	LST1	KRT18	COL3A1	TFF3	C1QC	C1S	SCGB3A1	HLA-DRA	HLA-DRA	COL1A1	PRF1	GZMB	FOS	IFI30
4	FCN1	KRT7	RARRES2	MMRN1	C1QB	COL6A2	WFDC2	HLA-DPA1	HLA-DQB1	BGN	GZMB	NKG7	IL6ST	FCER1G
5	COTL1	SPINT2	COL6A3	GNG11	APOE	COL1A1	PIGR	HLA-DQA1	HLA-DQA1	SPARC	FGFBP2	CD7	AKR7A2	LILRB2
6	FCER1G	EPCAM	BGN	RAMP2	TYROBP	COL1A2	AGR2	HLA-DQB1	CST3	COL6A2	CST7	CTSW	JUNB	COTL1
7	TYROBP	CLDN4	C1S	APP	APOC1	SERPING1	MUC5B	HLA-DRB1	HLA-DRB1	AEBP1	CTSW	GZMA	KRT18	PSAP
8	C5AR1	S100A6	IGFBP7	TFPI	FCER1G	COL3A1	CXCL17	HLA-DRB5	CD74	COL6A3	SPON2	PRF1	MGST1	TYROBP
9	PLAUR	C19orf33	NNMT	TIMP3	CD68	CALD1	SCGB1A1	GSN	HLA-DRB5	FN1	CD247	CST7	EGR1	FCGR3A
10	TIMP1	IFI27	SERPING1	ECSCR	LYZ	RARRES2	KRT19	CD83	HLA-DMA	DCN	KLRD1	CXCR4	WFDC2	CTSS
11	PSAP	TM4SF1	COL1A1	IGFBP4	FTL	IGFBP4	KRT7	SYNGR2	SPI1	MMP2	GZMH	KLRD1	RABL6	C5AR1
12	LILRB2	MDK	DCN	PPFIBP1	CTSB	COL6A1	CP	CST3	HLA-DMB	LUM	FCGR3A	CD8A	RTN3	SPI1
13	CTSS	TACSTD2	CALD1	TM4SF1	SPI1	TPM2	CLU	IFI30	TYROBP	CALD1	CCL5	ZFP36L2	FOSB	SERPINA1
14	CST3	S100A10	AEBP1	CAVIN2	GRN	FN1	CYB5A	SPI1	IFI30	C1S	GZMA	ZNF683	TIMP1	SMIM25
15	LILRA5	HSPB1	MMP2	FABP4	CST3	CCDC80	C3	VIM	AIF1	RARRES2	CCL4	GZMH	MICOS13	SAT1

Rank	OLR1+ Classical Monocyte	Pericyte	Plasma	Plasmacytoid Dendritic	Platelet/Megakaryocyte	Proliferating Basal	Proliferating Macrophage	Proliferating NK/T	Proximal Basal	Proximal Ciliated	Signaling Alveolar Epithelial Type 2	TREM2+ Dendritic	Vascular Smooth Muscle	Vein
1	IL1B	IGFBP7	MZB1	JCHAIN	CLU	H2AFZ	STMN1	HMGB2	SLPI	ADHFE1	SFTPA1	C1QC	IGFBP7	VWF
2	G0S2	SPARC	DERL3	GZMB	EFEMP1	H2AFV	TUBB	STMN1	KRT19	PTPRT	SFTPA2	APOE	MYL9	IGFBP7
3	SOD2	A2M	SSR4	PLD4	CALD1	TUBB	TUBA1B	MKI67	CXCL17	ARMC4	SFTPB	CTSB	TPM2	ACKR1
4	BCL2A1	RGS5	FKBP11	IRF7	CD151	KRT19	H2AFZ	TUBB	KRT17	ZSCAN18	NAPSA	C1QA	CALD1	PECAM1
5	PLAUR	CALD1	XBP1	LILRA4	MTRNR2L12	KRT8	HMGN2	TUBA1B	TACSTD2	MLIP-IT1	SLC34A2	FTL	TAGLN	EGFL7
6	NFKBIA	BGN	CD79A	GPR183	STAB2	JPT1	MKI67	HMGB1	ELF3	CCDC65	NPC2	C1QB	ACTA2	MGP
7	IFI30	HIGD1B	TXNDC5	IRF4	NTS	SPINT2	HMGB1	HMGN2	KRT8	LKAAEAR1	SLPI	CTSZ	BGN	CALCRL
8	CXCL8	PDGFRB	JCHAIN	IRF8	PKHD1L1	S100A16	HMGB2	H2AFZ	FXYD3	RFX3	WFDC2	CD68	MGP	HSPG2
9	FCER1G	COL4A2	SEC11C	ITM2C	ABI3BP	HMGB1	H2AFV	H2AFV	PRSS23	DUSP18	SFTPD	PSAP	SPARCL1	RNASE1
10	TYROBP	COX4I2	PRDX4	SLC15A4	VWF	ENO1	GRN	CORO1A	AQP5	AC108681.1	MUC1	IFI30	COL6A2	IGFBP4
11	FTH1	COL4A1	HSP90B1	TCF4	SOX13	TUBA1C	CTSZ	TYMS	WFDC2	FHAD1	SFTA2	APOC1	SPARC	RAMP3
12	S100A9	LHFPL6	HERPUD1	NR4A3	RMRP	KRT18	CAPG	NUSAP1	CLDN4	ANKRD66	CYB5A	TYROBP	C11orf96	RAMP2
13	IER3	SPARCL1	ITM2C	IL3RA	SNCA	STMN1	CD68	RRM2	CD9	AC005906.2	CTSH	LYZ	A2M	IFI27
14	LYZ	COL1A2	IGHG1	GRASP	TNS2	TUBA1B	VIM	HIST1H4C	PERP	RTCA-AS1	RNASE1	FCER1G	TPM1	ENG
15	EREG	NDUFA4L2	SSR3	TSPAN13	SH3D19	CKS1B	CTSB	TMPO	AGR2	SEC14L3	CXCL17	CTSD	SERPING1	TM4SF1

Supplementary Table 3. Top 15 genes in clustered T cell subsets.

Rank	C0-Th-SFTPB	C1-Th-IL7R	C2-Tc-RPS12	C3-Tc-NKG7	C4-Treg-FOXP3	C5-Tc-CCL5	C6-Tc-GZMB	C7-Tc-HSPA1A	C8-Tfh-CXCL13	C9-Th-S100A6
**1**	SFTPB	IL7R	RPS12	NKG7	FOXP3	CCL5	GZMB	HSPA1A	NR3C1	FTL
**2**	SCGB1A1	VIM	RPL28	CST7	IL32	HLA-B	CCL5	HSPA1B	CXCL13	HLA-DRA
**3**	WFDC2	ANXA1	RPL41	CCL5	BATF	NKG7	CD8A	DNAJB1	FKBP5	S100A6
**4**	HP	FTH1	RPL30	GZMK	TNFRSF4	HLA-C	ITGAE	HSP90AA1	SRGN	CD74
**5**	SCGB3A2	LMNA	RPS27	GZMH	LTB	HLA-A	CD7	HSPB1	SMAP2	IGLV1-51
**6**	XIST	LTB	RPS14	CCL4	SAT1	GAPDH	NKG7	HSPA8	SNX9	APOE
**7**	SCGB3A1	CCR7	RPS25	FGFBP2	TNFRSF18	CD8A	ALOX5AP	HSPH1	SARAF	IGKV3-20
**8**	ASS1	GPR183	RPS3	GZMA	CTLA4	B2M	ZNF683	BAG3	DUSP4	SFTPA1
**9**	S100A14	KLF2	EEF1A1	GNLY	TIGIT	GZMA	CXCL13	HSPE1	IL6ST	CST3
**10**	CXCL3	ZFP36L2	RPS28	TNFSF9	IL2RA	LAG3	PRF1	HSPA6	SPOCK2	IGLV1-40
**11**	SERPINA1	CDKN1A	RPL39	CD8A	TNFRSF1B	GZMB	CD63	DUSP1	ITM2A	RPS26
**12**	DSG2	FOS	JUND	CCL4L2	SPOCK2	CD3D	KLRD1	JUN	JUNB	IGKV4-1
**13**	IGLV4-69	AHNAK	RPL10	GZMM	HLA-A	ACTB	CTSD	TXNIP	GAPDH	SFTPB
**14**	AQP5	SLC2A3	RPLP2	CMC1	PMAIP1	TMSB4X	KRT86	HSP90AB1	IFITM1	IGHV3-23
**15**	CXCL14	ZFP36	RPS15A	CTSW	DNPH1	COTL1	CTSW	UBC	RNF19A	HLA-DRB1
